# The Effect of the Optogenetic Stimulation of Astrocytes on Neural Network Activity in an In Vitro Model of Alzheimer’s Disease

**DOI:** 10.3390/ijms252212237

**Published:** 2024-11-14

**Authors:** Elena V. Mitroshina, Elizaveta P. Kalinina, Alena I. Kalyakulina, Alexandra V. Teplyakova, Maria V. Vedunova

**Affiliations:** 1 Institute of Biology and Biomedicine, Lobachevsky State University of Nizhny Novgorod, 23 Gagarin Avenue, 603022 Nizhny Novgorod, Russia; 2 Institute of Information Technologies, Mathematics and Mechanics, Lobachevsky State University, 603022 Nizhny Novgorod, Russia; 3 Institute of Biogerontology, Lobachevsky State University, 603022 Nizhny Novgorod, Russia; 4 Federal Center of Brain Research and Neurotechnologies, Federal Medical-Biological Agency, 119330 Moscow, Russia

**Keywords:** Alzheimer’s disease, amyloidosis, astrocyte, neuronal network activity, optogenetic, multielectrode arrays

## Abstract

Optogenetics is a combination of optical and genetic technologies used to activate or, conversely, inhibit specific cells in living tissues. The possibilities of using optogenetics approaches for the treatment of epilepsy, Parkinson’s and Alzheimer’s disease (AD) are being actively researched. In recent years, it has become clear that one of the most important players in the development of AD is astrocytes. Astrocytes affect amyloid clearance, participate in the development of neuroinflammation, and regulate the functioning of neural networks. We used an adeno-associated virus carrying the glial fibrillary acidic protein (GFAP) promoter driving the optogenetic channelrhodopsin-2 (ChR2) gene to transduce astrocytes in primary mouse hippocampal cultures. We recorded the bioelectrical activity of neural networks from day 14 to day 21 of cultivation using multielectrode arrays. A single optogenetic stimulation of astrocytes at 14 day of cultivation (DIV14) did not cause significant changes in neural network bioelectrical activity. Chronic optogenetic stimulation from DIV14 to DIV21 exerts a stimulatory effect on the bioelectrical activity of primary hippocampal cultures (the proportion of spikes included in network bursts significantly increased since DIV19). Moreover, chronic optogenetic stimulation over seven days partially preserved the activity and functional architecture of neuronal network in amyloidosis modeling. These results suggest that the selective optogenetic activation of astrocytes may represent a promising novel therapeutic strategy for combating AD.

## 1. Introduction

Alzheimer’s disease (AD) is currently the most prevalent neurodegenerative disorder, primarily affecting the elderly population. The incidence of AD continues to rise as life expectancy increases globally. The pathogenesis of AD involves multiple pathways, with one of the most critical being the abnormal accumulation of β-amyloid (Aβ), a product of the sequential proteolytic cleavage of amyloid precursor protein (APP) by β- and γ-secretase enzymes [1]. Aβ oligomers accumulate in the brain parenchyma, forming extracellular amyloid plaques. The amyloidogenic hypothesis is widely considered the primary cause of AD. Additionally, AD is characterized by the hyperphosphorylation of tau protein, leading to the formation of neurofibrillary tangles [2,3]. Neuroinflammation, reactive astrogliosis, mitochondrial dysfunction, and oxidative stress are also prominent features observed in the AD brain [4].

The hallmark of AD in patients is a progressive decline in cognitive function and memory, primarily attributed to the loss of neurons and synapses in the hippocampal formation and related areas, leading to disruptions in neural network activity [5,6,7].

Beyond neurons, glial cells, particularly astrocytes, are also profoundly affected in AD. Astrocytes play a crucial role in maintaining brain metabolic homeostasis [8,9,10] by regulating the supply of metabolic substrates to neurons and other glial cells [11], maintaining extracellular ion homeostasis, and facilitating neurovascular interactions [8,12,13]. Astrocytes are integral to synaptic transmission, as they express receptors, neurotransmitter transporters, and ion channels, allowing them to participate in the integration of neural information [14]. They also regulate synaptic plasticity and neuronal excitability through the release of neuroactive substances known as gliotransmitters [15].

In AD, the functional activity of astrocytes is significantly impaired, with disruptions observed in calcium signaling, neurotransmitter clearance (particularly glutamate), gliotransmitter release, energy metabolism, etc. [9,16]. Recent research has underscored the pivotal role of astrocytes in driving neuroinflammation and oxidative stress in AD [17,18], as well as in the production and clearance of amyloid proteins [19]. Given the crucial role of astrocytes in AD pathogenesis, targeting these cells for therapeutic interventions is a promising approach. Such strategies could mitigate neuroinflammation and oxidative stress while supporting neural network functionality.

One emerging method of cellular modulation is optogenetics—a modern technique that combines optical, genetic, and electrophysiological methods to regulate cellular activity using light-sensitive proteins [20]. Optogenetic tools offer the potential to modulate various biological processes within the cell, including signal transmission, protein expression and oligomerization, and gene expression regulation [21].

In most studies, optogenetic tools have primarily been utilized to regulate neuronal activity, examine the roles of different neuronal populations in certain behavior, and regulate sleep/wakefulness cycles. Moreover, there have been ongoing attempts to treat epilepsy and other neurological diseases using this approach [22]. However, within the past 5–8 years, successful examples of applying optogenetic techniques to astrocytes have emerged [22,23,24,25]. In astrocytes, an activation of the optogenetic ion channel ChR2 can induce depolarization, a decrease in intracellular pH, and an increase in the intracellular calcium concentration ([Ca^2+^]). These effects can modulate astrocytic activity and regulate the release of gliotransmitters [26].

Few studies have explored the potential of optogenetics for addressing Alzheimer’s disease (AD). Recent work has demonstrated that the optogenetic activation of hippocampal neurons in an AD model improves short-term, though not long-term, memory, while also attenuating neuroinflammation and providing neuroprotective effects in the central region of ChR2 expression [27]. Wang et al. showed that the use of the AAV5-CaMK-CHR2-mCherry construct for optical stimulation of glutamatergic neurons improved short-term memory. This improvement was likely mediated through the activation of GluR2 and mGluR5, increased expression of the NR subunit of the NMDA receptor, synapsin, and NeuN, and a concomitant reduction in the expression of GFAP and IL-6 [24].

In the present study, we investigated the effects of the optogenetic stimulation of astrocytes using the AAV-GFAP-ChR2-eYFP-WPRE construct on the bioelectrical activity of primary mouse hippocampal cell cultures in a model of β-amyloidosis. This research aims to deepen our understanding of AD mechanisms and explore potential therapeutic approaches for the treatment of this disease.

## 2. Results

### 2.1. The Effect of Optogenetic Stimulation of Astrocytes on Spontaneous Bioelectrical Activity of Neural Networks in Primary Hippocampal Cultures

**Figure 1:**
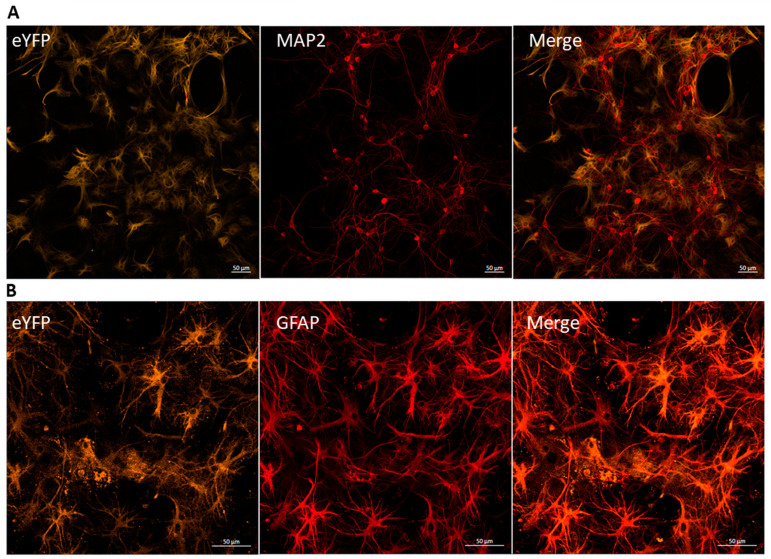
Representative immunocytochemical staining of a hippocampal cell culture infected with the AAV-hGFAP-ChR2-EYFP viral vector at 14 DIV. (**A**)—the orange channel shows eYFP fluorescence in cells transfected with the viral vector, and the red channel indicates marks neurons using MAP2. (**B**)—the orange channel shows eYFP fluorescence in cells transfected with the viral vector, and the red channel indicates astrocytes marked by GFAP. Images were captured using a Zeiss LSM 800 confocal microscope. Scale 50 µm.

Immunocytochemical labeling was performed to confirm the efficiency and specificity of transduction in the primary hippocampal cell cultures. The transduction efficiency was determined to be greater than 90% (Figure 1). Figure 1 demonstrates that the fluorescence of the viral protein eYFP was not colocalized with the neuronal marker MAP2 (Figure 1A) and was highly colocalized with the glial fibrillary acidic protein (GFAP, astrocytic marker) (Figure 1B), confirming the specificity of the viral construct’s expression.

Next, we examined the spontaneous bioelectrical activity of neural networks in primary hippocampal cultures under acute and chronic optogenetic stimulation of astrocytes.

Traditionally, a network burst is defined as an event where at least four spikes are recorded from different electrodes within a 50 ms interval [28,29,30]. However, bursts can vary significantly in their quantitative properties, making it useful to distinguish between the two classes of bursts—small bursts (containing 4 to 100 spikes within 50 ms) and large bursts (exceeding 100 spikes within 50 ms). This classification captures the predominant functional activity of the network. Identifying large network bursts allows for a more detailed analysis of network structure using cross-correlation methods, enabling the visualization of functional network architecture through raster plots.

Our group has previously demonstrated that the formation and maturation of neural network activity are closely linked to the progressive establishment of new synaptic contacts between neurons and the development of synapses. The initial network bursts in hippocampal cultures typically appear around DIV7, characterized by a relatively small number of spikes. As cultivation progresses, the number of spikes in large bursts increases, and by DIV14, with the maturation of synaptic contacts, a stabilization of bioelectrical parameters is observed [30]. Therefore, we initiated optogenetic stimulation of astrocytes in hippocampal cultures at DIV14, when stable neural network activity had formed.

An analysis of electrophysiological data indicated that a single optical stimulation of AAV-hGFAP-ChR2-EYFP-transduced cultures at DIV14 did not significantly alter bioelectrical activity. However, chronic daily optical stimulation from DIV14 to DIV21 modulated the spontaneous bioelectrical activity of primary hippocampal cultures, with changes becoming evident on the fifth or sixth day of stimulation (DIV18–19).

**Figure 2:**
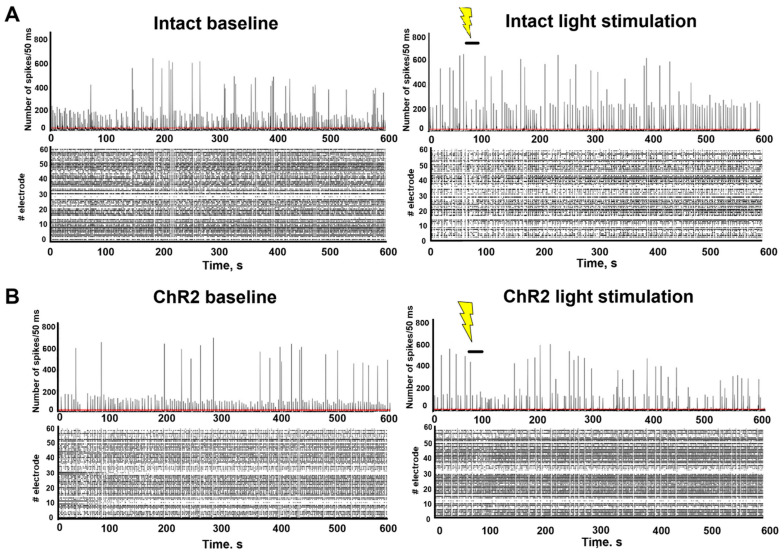
Number of spikes/50 ms and raster diagrams of spontaneous bioelectrical activity in primary hippocampal cultures during astrocytes optogenetic stimulation in vitro (DIV14): (**A**) intact culture; (**B**) AAV-hGFAP-ChR2-EYFP-transduced culture. Red line—threshold, yellow lightning—light stimulation 470 nm.

**Figure 3:**
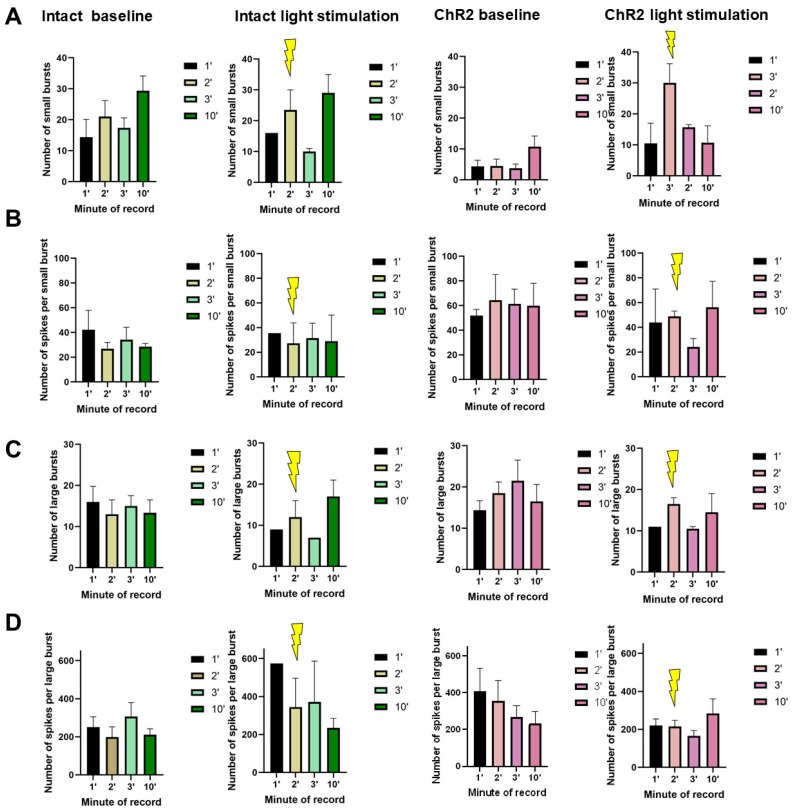
Main parameters of spontaneous bioelectrical activity in primary hippocampal cell cultures on DIV 14: (**A**) number of small bursts per minute; (**B**) number of spikes per small burst; (**C**) number of large bursts per minute; (**D**) number of spikes per large burst. Yellow lightning—light stimulation 470 nm. M ± SEM. No statistically significant differences vs. DIV14 according to the Kruskal–Wallis test.

Figure 2 presents raster plots of spontaneous bioelectrical activity at DIV14, and Figure 3 illustrates the main parameters of bioelectrical activity during a minute-by-minute analysis of recordings made during optogenetic stimulation. Optical stimulation began at the 61st second of recording and lasted for 30 s. No significant short-term changes in neural network activity were observed, though a trend towards a decrease in the number of spikes in both small and large bursts was noted one minute after photostimulation in AAV-hGFAP-ChR2-EYFP-transduced cultures. By the 10th minute of recording, the number of spikes in the bursts had returned to baseline values.

In the subsequent days of cultivation (up to DIV21), optical stimulation similarly did not induce significant immediate changes in the primary parameters of bioelectrical activity during a ten-minute recording session (Supplementary Materials, Figures S1 and S2).

**Figure 4:**
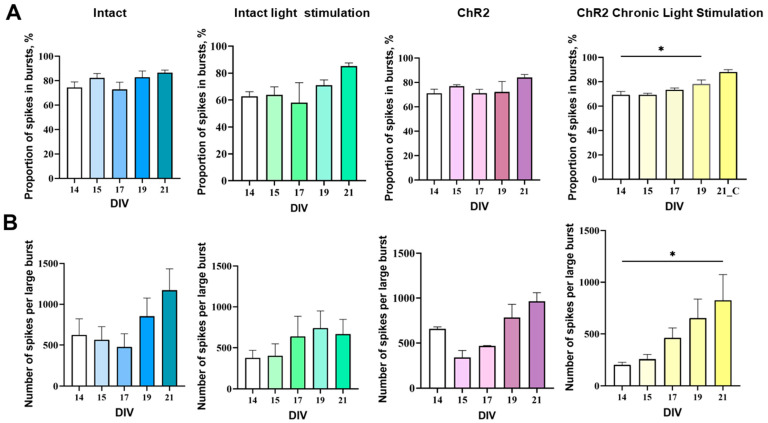
Main parameters of spontaneous bioelectrical activity in primary hippocampal cell cultures on DIV 14. (**A**) Proportion of spikes in bursts, %; (**B**) number of spikes per large burst. M ± SEM. * *p* < 0.05—the differences are significant vs. DIV14 according to the Kruskal–Wallis test.

Chronic optogenetic stimulation was found to exert a stimulatory effect on the baseline bioelectrical activity of the primary hippocampal cultures. After seven sessions of daily optogenetic stimulation (by DIV21), the proportion of spikes included in the network bursts significantly increased compared to the values recorded at DIV14 (“ChR2 Chronic Light” DIV14: 69.26 ± 2.85%, DIV19: 78.17 ± 3.34%), indicating a more complex functional profile of network burst activity. In the intact group, no significant changes in the proportion of spikes in bursts were observed throughout the observation period (Figure 4).

Despite accumulating evidence suggesting the potential involvement of hypoxia and/or altered responses to hypoxia in the pathogenesis of Parkinson’s disease, the precise nature of these relationships remains incompletely understood.

In the “ChR2 Chronic Light” group, the number of spikes in the large bursts on day 21 of cultivation was significantly (4.08 times) higher than on day 14. In contrast, in the “Intact” group and “ChR2” group with single stimulation, the number of spikes in the large bursts on DIV21 increased by only 1.61 and 1.88 times, respectively, compared to DIV14.

No significant changes were observed in other parameters, including the number of small and large bursts, the duration and amplitude of large and small bursts, and the number of spikes in the small bursts. The corresponding data are presented in the Supplementary Materials (Figure S3).

**Figure 5:**
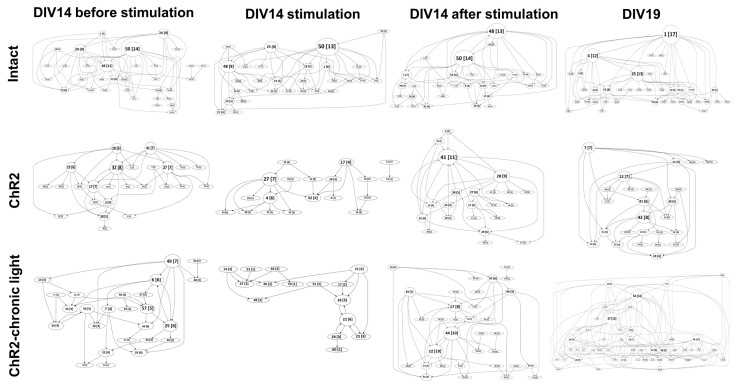
Functional structure of neural networks in the primary hippocampal cultures in β-amyloidosis modeling. Graphical representation of the correlated connections among neurons in the network. The number of the corresponding electrode is indicated in the circles. The number of connections on the electrode is indicated in square brackets. The vertex size is proportional to the number of significant connections. To enable a more detailed examination of this figure, in the Supplementary Materials each graph is presented as a separate, enlarged figure.

An analysis of the rasters showing the functional connections between the groups of neurons localized on the electrodes of the multielectrode array revealed a trend towards a simplified functional network during and in the first minute after optogenetic stimulation. The number of hubs (key nodes of network connectivity) decreased, as did the number of connections between them. However, by the tenth minute after optogenetic stimulation, the complexity of the network had returned to its initial level (Figure 5). Notably, no significant changes in the hub coefficient were detected.

Thus, we have demonstrated that a single optogenetic stimulation of astrocytes from primary hippocampal cultures transduced with the AAV-hGFAP-ChR2-EYFP vector does not produce significant effects on neural network activity in either short-term or long-term observation periods. However, chronic optogenetic stimulation has a modulating effect, increasing the baseline level of bioelectrical activity at DIV21 and enhancing the complexity of the neural network structure.

### 2.2. Effect of Optogenetic Stimulation of Astrocytes on Neural Network Activity of Primary Hippocampal Cultures in β-Amyloidosis Modeling

**Figure 6:**
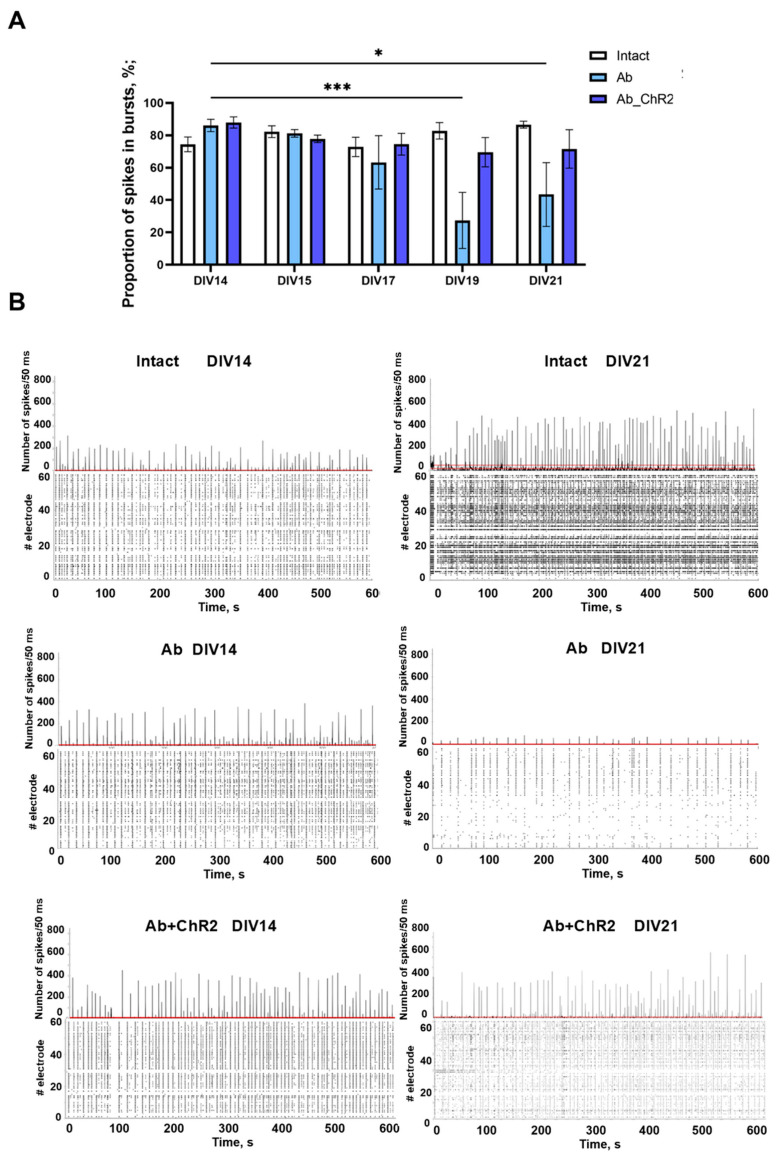
Bioelectrical activity of primary hippocampal cell cultures during optogenetic stimulation of astrocytes transduced with AAV-hGFAP-ChR2-EYFP in β-amyloidosis modeling: (**A**) proportion of spikes in bursts, %; (**B**) number of spikes/50 ms and raster diagrams of spontaneous bioelectrical activity. Red line—threshold. M ± SEM. * *p* < 0.05; *** *p* < 0.001—the differences are significant vs. DIV14 according to the Kruskal–Wallis test.

Our previous work has shown that β-amyloidosis disrupts neural network formation [31]. The dynamics of the bioelectrical activity of cultures exposed to β-amyloid application confirmed our previous results. The suppression of bioelectrical activity occurred gradually. Starting from the 19th day of cultivation, a significant decrease in the proportion of spikes included in network bursts was observed in the “Ab” group (DIV14: 86.1 ± 3.78%; DIV19: 27.38 ± 17.32%; DIV21: 43.44 ± 19.71%) (Figure 6).

**Figure 7:**
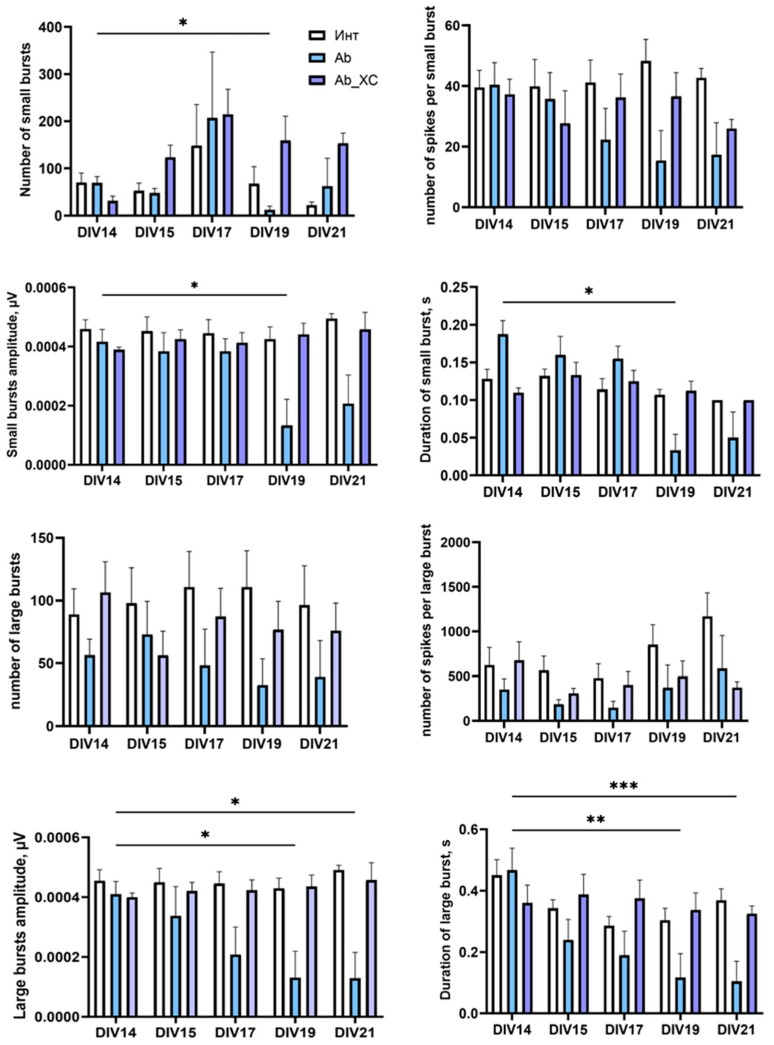
Main parameters of bioelectrical activity of primary hippocampal cell cultures during optogenetic stimulation of astrocytes transduced with AAV-hGFAP-ChR2-EYFP in β-amyloidosis modeling. M ± SEM. * *p* < 0.05; ** *p* < 0.01; *** *p* < 0.001—the differences are significant vs. DIV14 according to the Kruskal–Wallis test.

Moreover, the modeling of β-amyloidosis by DIV19 resulted in a marked reduction in several parameters, including the number of small bursts, their amplitude, and their duration. Additionally, there was a clear trend towards a decrease in the number of large bursts, alongside a statistically significant reduction in their duration and amplitude (Figure 7). Notably, by day 19, a significant proportion of cultures (40%) in the “Ab” group completely lacked burst activity, with only isolated spikes being recorded.

Compared to the baseline level in the “Ab” group, the number of large network bursts decreased by 1.72 times with DIV19, and their amplitude and duration decreased by 3.12 and 4 times, respectively. Daily optogenetic stimulation of primary hippocampal cell cultures transduced with AAV-hGFAP-ChR2-EYFP contributed to the preservation of the main parameters of spontaneous bioelectrical activity throughout the observation period. In the “Ab + ChR2” group, the studied parameters did not differ significantly from the baseline levels.

**Figure 8:**
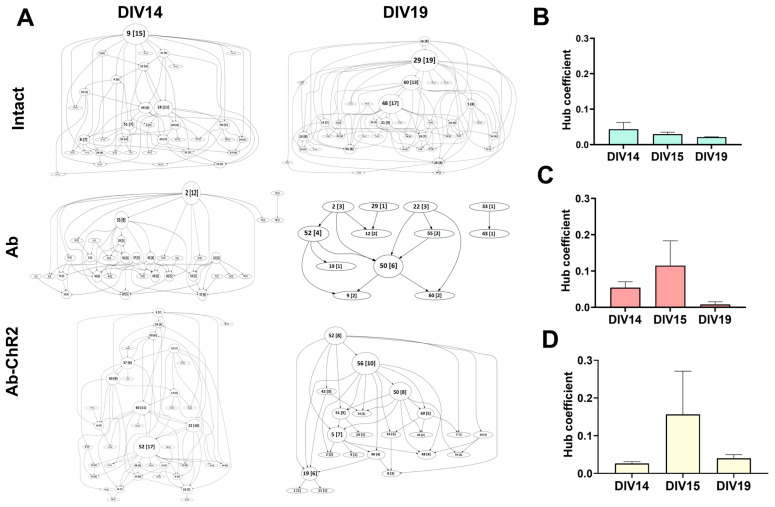
Functional structure of neural networks in the primary hippocampal cultures in β-amyloidosis modeling. (**A**) Graphical representation of the correlated connections among neurons in the network. The number of the corresponding electrode is indicated in the circles. The number of connections on the electrode is indicated in square brackets. The vertex size is proportional to the number of significant connections. To enable a more detailed examination of this figure, in the Supplementary Materials each graph is presented as a separate, enlarged figure. (**B**–**D**) The dynamics of the hub coefficient in β-amyloidosis modeling: (**B**) “Intact” group, (**C**) “A” group, (**D**) “Ab-ChR2 Chronic Light Stimulation” group. M ± SEM. No statistically significant differences vs DIV14, according to the Kruskal–Wallis test.

The construction of directed graphs to evaluate the functional architecture of the network revealed that in the “Intact” group, the graph diameter significantly increased, indicating an expansion in the number of nodes and connections for DIV19 compared to DIV14, with a concurrent increase in the number of hubs. Hubs represent key functional nodes in the network with a large number of connections. Simultaneously, throughout cultivation in the intact group, the hub coefficient, which reflects the number of connections associated with the hub node, gradually decreased. This combination of an increased number of hubs and a moderate (no more than 1.9-fold) decrease in the hub coefficient suggests an increase in network complexity and distribution (Figure 8).

Amyloidosis modeling resulted in a simplification of the functional structure of the neural network in the primary hippocampal cultures, reducing the number of hubs. Our findings indicate that amyloidosis leads to a decrease in the graph diameter, reflecting a reduction in the number of nodes and connections for DIV19 compared to DIV14. Simultaneously, the hub coefficient in the “Ab” group decreased more than 5-fold with DIV19.

Daily optogenetic stimulation of astrocytes partially mitigated neurodegenerative changes in the neural network structure (Figure 8). In the “Ab + ChR2” group, a relatively well-developed network structure with several key nodes was preserved. For DIV19, the hub coefficient was comparable to the values observed at DIV14.

**Figure 9:**
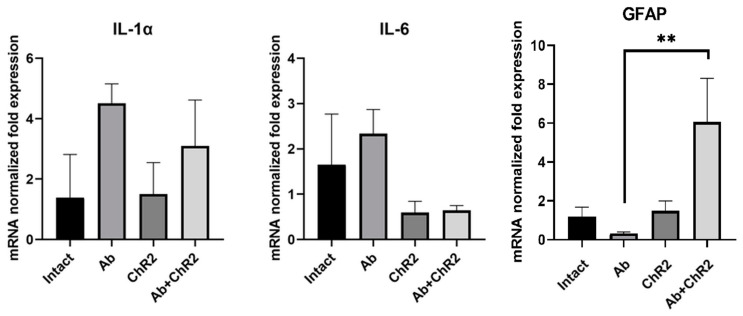
Level of mRNA expression of genes of interest in hippocampal cultures after chronic optogenetic stimulation of astrocytes on DIV21. ** *p* ≤ 0.01 (one-way ANOVA and Tukey’s multiple post hoc test).

The changes in the functional activity of neuron-glial networks during optogenetic stimulation and AD modeling are apparently related to the transition of astrocytes into a reactive state and changes in the synthesis of various biologically active substances, particularly proinflammatory cytokines. Following our hypothesis, we examined the mRNA expression levels of IL-1a and IL-6 proinflammatory cytokines and GFAP as reactive markers on DIV21. The RT-PCR data are provided in Figure 9.

We have demonstrated that chronic optogenetic stimulation increased the expression of GFAP and that there was a tendency toward a decrease in proinflammatory cytokines IL-1a and IL-6 in the “Ab + ChR2” group compared to the “Ab” group.

Thus, the protocol developed for the optogenetic stimulation of astrocytes in primary hippocampal cultures transduced with AAV-hGFAP-ChR2-EYFP effectively mitigates neurodegenerative processes in neural networks, maintaining the bioelectrical activity of primary hippocampal cultures and partially preserving the functional architecture of the neural network.

## 3. Discussion

One of the most well-established hypotheses regarding the development of Alzheimer’s disease (AD) is the β-amyloid cascade hypothesis. Despite extensive efforts to preserve neuronal function during the progression of AD, success has been limited. Recently, astrocytes have emerged as a potential therapeutic target in AD treatment. Similarly to neurons, astrocytes are involved in the synthesis and processing of β-amyloid, and even minor alterations in the level of Aβ production by astrocytes can significantly contribute to the overall amyloid burden [32]. Moreover, the cascade of pathological events associated with AD includes neuroinflammation, with astrocytes playing a key role in its development within nervous tissue. It is essential to note that astrocytes have a dual role in inflammation: they can both promote inflammatory processes through the activation of the NF-κB pathway and the production of nitric oxide (NO), reactive oxygen species (ROS), and proinflammatory cytokines such as IL-1β, IL-6, and TNF, and simultaneously release anti-inflammatory and neuroprotective factors [9,10]. Astrocytes exert a profound influence on neuronal survival, synaptic transmission, calcium buffering, nutrient transport, and neurotransmitter release [9,33,34]. Therefore, regulating the functional state of astrocytes appears to be a promising strategy for preventing and mitigating neurodegenerative processes. In this study, we explored the potential of modulating neural network activity through astrocyte activation using optogenetic tools.

Optogenetics has revolutionized the field by enabling researchers to modulate neuronal activity, intracellular signaling pathways, or gene expression with high spatial and temporal precision through the selective expression of exogenous light-sensitive opsin proteins [9,35]. Although optogenetic technologies are rapidly evolving, their use has primarily been focused on neurons. In astrocytes, the activation of ChR2 can induce depolarization, a decrease in intracellular pH, and an increase in [Ca^2+^], thereby modulating astrocyte activity and the release of gliotransmitters [26,36]. However, the effects of optogenetic stimulation of astrocytes on neural network bioelectrical activity remain largely unexplored.

To investigate the impact of astrocyte activation on the activity of neural networks in primary hippocampal cultures, we utilized the AAV-GFAP-ChR2-eYFP construct. A single optogenetic stimulation at DIV14 did not result in significant changes in neural network activity. Nonetheless, a transient trend toward a reduction in the number of spikes in both large and small bursts of impulses was observed within one minute of ChR2 channel activation in astrocytes. Additionally, a short-term simplification of the functional architecture of the network was detected, characterized by a reduction in the graph diameter, which reflects the functional connections between neuronal groups, and a decrease in the number of hubs. These alterations were transient and returned to baseline levels by the 10th minute following optical stimulation.

These results align with the existing literature demonstrating that the stimulation of astrocytic ChR2 with light results in the depression of basal field excitatory postsynaptic potentials (fEPSPs) in the hippocampal CA1 region [37]. Since astrocytes expressing ChR2 in primary hippocampal cultures are capable of releasing both “activating” and “inhibitory” gliotransmitters that modulate synaptic transmission, it is plausible that light stimulation leads to the transient release of a substantial quantity of inhibitory gliotransmitters [37]. Additionally, it has been shown that ChR2-expressing astrocytes can release ATP rapidly enough to excite CCK(+) interneurons via P2Y1 receptors, which enhances GABA release and increases synaptic inhibition in neural networks [38]. This mechanism could positively impact neuronal viability in AD, as it may prevent the onset of excitotoxicity. Hyperactive glutamatergic signaling is observed in the early stages of AD, often associated with insufficient clearance of glutamate in the extracellular space. When astrocytes interact with amyloid, they lose their ability to effectively absorb glutamate, which impairs synapse function [39]. Optogenetic stimulation of astrocytes could potentially mitigate the excessive activation of glutamatergic neurons and prevent excitotoxicity through GABA-mediated inhibition.

Interestingly, our study demonstrated that chronic daily optogenetic stimulation led to an increase in the baseline level of neural network bioelectrical activity by day 19 of culture. Specifically, the proportion of spikes that were part of burst events significantly increased after five days of daily stimulation. This effect may be due to cumulative changes in protein synthesis during astrocyte activation. It is well-established that astrocytes actively participate in activity-induced molecular rearrangements within neural circuits and secrete various mediators that modulate neuronal metabolism, enhancing the transcription and translation of genes involved in neural plasticity [40,41,42].

The second part of the experimental work focused on the reorganization of neural networks under conditions modeling amyloidosis, a key pathogenetic component of AD. To date, changes in spontaneous bioelectrical activity in AD neural networks have been insufficiently studied. Most electrophysiological data have been obtained through patch-clamp methods, which focus on synaptic transmission in individual neurons and synaptic terminals [43,44]. Multichannel electroencephalography studies have only indirectly characterized neural network activity in AD models [6,45]. Our use of multielectrode arrays provided novel insights into the impact of astrocytes on neural network activity in β-amyloidosis.

Consistent with our previous findings on both bioelectrical [31] and calcium activity [31,46], we observed significant disruptions in spontaneous bioelectrical activity in neural networks of primary hippocampal cultures subjected to chronic application of Aβ. In the modeling of chronic beta-amyloidosis, a significant decrease in nearly all assessed parameters was observed, including the proportion of spikes within network bursts relative to the total number of spikes recorded, as well as the amplitude and duration of both small and large bursts, beginning from DIV19. Moreover, in a substantial proportion of cultures (40%), burst activity was entirely abolished, with only individual spikes remaining.

Subsequently, we evaluated the effect of optogenetic stimulation of astrocytes on neural network functionality. Following 30 s of daily optogenetic stimulation over 7 days (from DIV14 to DIV21), none of the evaluated bioelectric parameters showed significant deviations from the control values. The optogenetic stimulation of astrocytes successfully maintained a high level of neural network activity. Cross-correlation analysis and the construction of neural network graphs further demonstrated that chronic optogenetic stimulation of astrocytes preserved both the level of functional interactions and the overall functional architecture of the network.

Research into the application of optogenetics for the correction of neurodegeneration is in its early stages. One of the pioneering studies in this area was conducted by Iaccarino et al., which demonstrated that selective optogenetic stimulation of fast-firing parvalbumin-positive (FS-PV) interneurons at gamma frequencies (40 Hz), but not at other frequencies, significantly reduced amyloid-β (Aβ) 1–40 and Aβ 1–42 isoforms [47]. Additional studies have similarly shown that optogenetic activation of hippocampal neurons improved short-term, but not long-term, memory in AD models [24,27,48]. These studies also noted an increase in synaptic density and plasticity, as well as astrocyte activation. Importantly, chemogenetic inhibition of astrocytes negated the effects of neuronal optostimulation, indicating that astrocyte activation is critical for memory recovery [48]. Furthermore, Lee et al. demonstrated that the optogenetic activation of astrocytes restored non-REM (NREM) slow waves in sleep disorders, reduced amyloid deposition, prevented neuronal calcium overload, and improved memory performance in APP/PS1 mice [49]. The authors attributed these effects to normalized inter-astrocytic calcium signaling, which in turn mitigated neuronal calcium overload, a known contributor to sleep and memory deficits in AD.

Our study builds on this emerging body of knowledge. The preservation of spontaneous bioelectrical activity in primary hippocampal cell cultures under optogenetic stimulation in a β-amyloidosis model suggests that such stimulation may slow disease progression.

Our future research will focus on elucidating the neuroprotective mechanisms underlying the optogenetic stimulation of astrocytes, with particular emphasis on their inflammatory and anti-inflammatory profiles. It has been demonstrated that the photostimulation of astrocytes can protect neurons from apoptosis and enhance neurobehavioral outcomes in stroke-affected rats compared to control groups. Moreover, optogenetically activated astrocytes exhibited a significantly increased expression of the anti-inflammatory cytokine Il-10, suggesting that these astrocytes were activated towards the neuroprotective A2 phenotype [25]. Furthermore, an increase in the mRNA expression of GFAP and decrease in the level of IL-1a and IL-6 proinflammatory cytokines expression, as observed in our study, may indicate that neuroinflammatory processes are modulated during optogenetic stimulation. These changes reflect long-term metabolic shifts, which likely explain the lack of short-term effects of optogenetic stimulation in the amyloidosis model. The protective effects only become evident after chronic light stimulation

Additionally, the duration of the positive effects of optogenetic stimulation remains unknown, warranting further experimentation to explore the longevity and potential of this approach.

The practical application of optogenetic methods in treating neurological and neurodegenerative diseases entail certain challenges. One of the primary limitations is the invasiveness of optogenetics, stimulating nerve cells currently requires implanting optical fibers into the brain to deliver a light stimulus. Athough infrared radiation, which penetrates biological tissues more deeply, could offer a potential solution, its application is restricted by the absorption spectra of opsins. Currently, the opsins used in optogenetics are activated by light in the blue or green spectrum.

Although this is a substantial obstacle, several research groups have proposed innovative solutions. For example, upconversion nanoparticles have been developed that can absorb near-infrared (NIR) light and emit visible blue-green light, enabling the stimulation of transfected neurons in deeper brain layers [50]. While the current efficiency of NIR-to-blue light conversion is low, future advancements may improve this technique, potentially enabling optogenetic applications without the need for surgical optical fiber implants.

Furthermore, Gong et al. have developed a highly photosensitive opsin, SOUL, capable of activating neurons in deep brain regions through transcranial optical stimulation, which has successfully induced behavioral changes in mice. This method was also proven to be effective in macaque models. The study underscores the high specificity and temporal precision of SOUL, presenting it as a promising tool for the non-invasive optostimulation of deep brain structures [51].

Additionally, we emphasize that our study has demonstrated the feasibility of preserving neural network functionality through the metabolic stimulation of GFAP-positive astrocytes, which can be achieved via various agents, including pharmacological interventions.

## 4. Materials and Methods

### 4.1. Ethic Statement

The object of the study was primary hippocampal cell cultures obtained from C57BL/6 mouse embryos on the 18th day of gestation. All experimental procedures were carried out in accordance with Act 708n (23 082010) of the Russian Federation National Ministry of Public Health, which states the rules of laboratory practice for the care and use of laboratory animals, and Council Directive 2010/63 EU of the European Parliament (22 September 2010) on the protection of animals used for scientific purposes, and were approved by the Bioethics Committee of Lobachevsky State University of Nizhny Novgorod (protocol No. 14 dated 19 January 2018).

### 4.2. Obtaining and Culturing Primary Hippocampal Cell Cultures

On the 18th day of gestation, the pregnant mice were euthanized by cervical dislocation. The uterus containing the embryos and the extracted embryos were placed in sterile Hanks’ solution (PanEco, Moscow, Russia). The hippocampi were surgically isolated, transferred to phosphate-buffered saline (PBS, Thermo Fisher Scientific, Waltham, MA, USA), and mechanically dissociated. The tissue was then treated with 0.25% trypsin-EDTA solution (Thermo Fisher Scientific, USA) for 20 min at 37 °C. The cells were then washed with PBS and resuspended in Neurobasal Medium™ (Thermo Fisher Scientific, USA), supplemented with 5% fetal bovine serum (FBS, Biosera, Cholet, France), 1% B27 supplement (Thermo Fisher Scientific, USA), and 0.5% L-glutamine (Thermo Fisher Scientific, USA). The resulting cells were seeded onto MEA60 multielectrode arrays (Multichannel Systems, Reutlingen, Germany) at a density of 8500–9000 cells/mm^2^. Prior to seeding, the array’s electrodes were coated with polyethyleneimine solution (1 mg/mL, Sigma-Aldrich, Schnelldorf, Germany) and subsequently washed with PBS. The cultures were maintained in a CO_2_ incubator at 37.5 °C with constant humidity. For medium changes, Neurobasal Medium™ (Thermo Fisher Scientific, USA) with 1% FBS, 1% B27, and 0.5% L-glutamine was used [30].

### 4.3. Constructs and Transduction of Primary Hippocampal Cell Cultures

The study employed the following groups of cultures:

- “Intact”—cultivated for 21 days according to the standard protocol without additional interventions (*n* = 6).
- “Intact Light”—intact cultures exposed to a single optical stimulation on DIV14 (*n* = 3).
- “ChR2”—cultures transduced on DIV10 with the viral vector AAV-GFAP-ChR2-eYFP-WPRE, followed by a single optical stimulation on DIV14 (*n* = 3).
- “ChR2 Chronic Light Stimulation”—cultures transduced with AAV-GFAP-ChR2-eYFP-WPRE on DIV10 and subjected to daily optical stimulation from DIV14 to DIV21 (*n* = 3).
- “Ab”—cultures exposed to Aβ 1–42 at a concentration of 3.5 μM with each medium change from DIV10 to DIV21, modeling β-amyloidosis (*n* = 5).
- “Ab-ChR2 Chronic Light Stimulation”—cultures subjected to β-amyloidosis modeling from DIV10 to DIV21, transduced with AAV-GFAP-ChR2-eYFP-WPRE, and exposed to daily optical stimulation from DIV14 to DIV21 (*n* = 5).

### 4.4. Viral Vectors and Transduction of Primary Hippocampal Cell Cultures

The AAV-GFAP-ChR2-eYFP-WPRE viral vector was used for selective expression in astrocytes. This vector was generated based on the pAAV-GFAP-ChR2-EYFP plasmid.

Transduction of primary hippocampal cultures was performed on DIV10. A volume of 100 μL of the culture medium was collected, and 1 μL of the viral preparation (corresponding to a viral load of 2.5 × 10^9^ genomic copies) was added. The cultures were incubated for 10 min, after which the medium was returned to its original volume (1 mL).

### 4.5. Recording of Spontaneous Bioelectrical Activity and Processing of Electrophysiological Data

**Figure 10:**
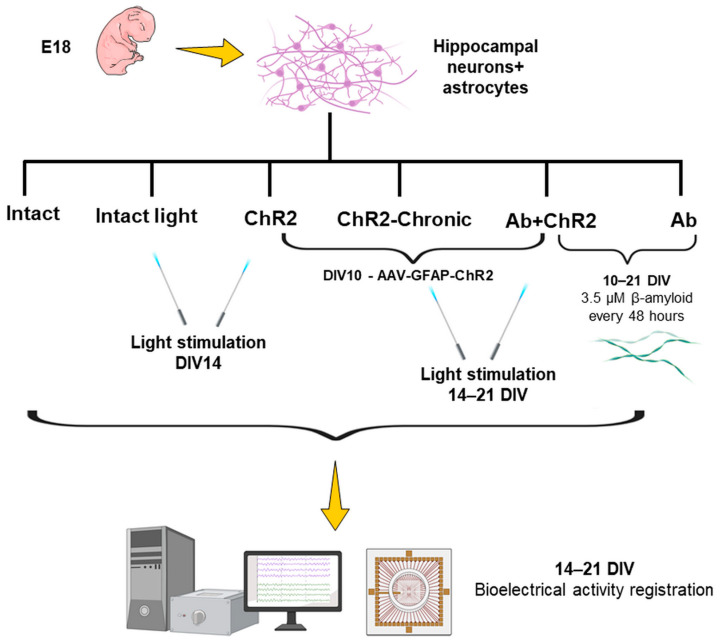
Scheme of experiment.

Spontaneous bioelectrical activity of primary hippocampal cell cultures was recorded at DIV14, DIV15, and then every 48 h until DIV21 using the MEA2100-2x60-System-E multielectrode setup (Multichannel Systems, Germany). The recording duration was set to 10 min. In the “Intact-light”, “ChR2”, “ChR2 Chronic Light”, and “Ab-ChR2 Chronic Light” groups subjected to optogenetic stimulation, baseline activity was first recorded for 10 min. Following this, the stimulation was recorded, with the culture being exposed to photo exposure from 60 to 90 s (Figure 10).

Electrophysiological data were processed using the custom-made software MEAxtd developed on Phyton (version 1.0). The parameters of spontaneous bioelectrical activity analyzed included the average number of bursts, the average number of spikes within bursts, burst duration, burst amplitude, and the proportion of spikes within bursts relative to the total number of spikes in the recording [29,52]. A custom software algorithm was employed to classify network events into small bursts (less than 100 spikes in 50 ms) and large bursts (more than 100 spikes in 50 ms).

To analyze the neural network architectonics, directed graphs of network bursts were generated using a custom algorithm. Initially, spikes synchronous with delays between electrodes were identified; since bursts are detected on multiple electrodes simultaneously, the delay in signal transmission from one electrode to another must be accounted for. For all electrode pairs involved in network bursts, 50 intervals with increasing durations from 1 to 50 ms (in 1 ms increments) were examined. For each interval, the number of spikes that were synchronous with a delay was calculated (i.e., the number of spikes with delays of 1 ms, 2 ms, 3 ms, and so on, up to 50 ms for each electrode pair). During the analysis, a pair of electrodes, denoted as m and n, was considered, where the signal passes from electrode m to electrode n. For each interval (1 ms, 2 ms, etc., up to 50 ms), the ratio of the number of spikes synchronous with a delay between these electrodes to the total number of spikes on electrode n was computed. These ratios were calculated for all electrode pairs, resulting in a ranking based on the strength of their connectivity. To ensure only the strongest connections are included in the graph, the top 5% of electrode pairs based on the calculated ratio values were selected from the ranked list. For these selected pairs, a directed graph was constructed, where the nodes represent individual electrodes, and the edges represent connections between them, indicating the presence of synchronous spikes with delays between the electrodes. The size of each node corresponds to the number of connections associated with the respective electrode, and the weight of each edge reflects the time delay between synchronous spikes.

### 4.6. Optical Stimulation of Primary Hippocampal Cell Cultures

For optogenetic stimulation, the Thorlabs optogenetic kit (Thorlabs Inc., Newton, NJ, USA) was used. An optical fiber with a nozzle was positioned using a precision micromanipulator, with the nozzle immersed in the medium directly above the cells on the microelectrodes. Stimulation was conducted using a diode emitting light at a wavelength of 470 nm. Stimuli were delivered at 100% diode power for a duration of 30 s.

### 4.7. Modeling of β-Amyloidosis as a Key Pathogenetic Component of AD

To prepare a 1 mM solution of fibrillar Aβ 1–42, 222 μL of hexafluoro-2-propanol (HFIP) was added to a vial containing 1 mg of lyophilized Aβ 1–42 (InnovaGen, Lund, Sweden). The solution was incubated for 30 min at room temperature, then transferred to a separate container and allowed to dry in a laminar flow hood until a transparent film formed. To create a 5 mM solution, 44.3 μL of dimethyl sulfoxide (DMSO) was added to 1 mg of amyloid and mixed for 30 s. Following this, 208 μL of 10 mM hydrochloric acid were added, and the mixture was incubated at 37 °C for 24 h to obtain the fibrillar form of amyloid.

Starting from the 10th day of cultivation, fibrillar Aβ 1–42 was added to the cultures in the “Ab” and “Ab-ChR2 Chronic Light” groups at a concentration of 3.5 μM during each medium change to model amyloid pathology [31,46].

### 4.8. Immunocytochemistry

Prior to immunocytochemical staining, the cultures were washed three times with PBS and then fixed in a solution containing 4% paraformaldehyde (PFA) and 15% sucrose. Fixation was carried out for 15 min in a CO_2_ incubator at 37 °C with 5% CO_2_. The immunocytochemical staining procedure followed this protocol: non-specific binding was blocked for 1 h using a blocking buffer consisting of 5% FBS, 0.5% Triton X-100, and 0.1% Tween 20 in 1x PBS. Primary antibodies, diluted 1:500, were applied to the cultures and incubated for 3 h at +4 °C. The cultures were washed three times with wash buffer (0.1% Triton X-100, 0.1% Tween 20 in 1x PBS) and then incubated for 90 min with secondary antibodies at a dilution of 1:1000. The following antibodies were used: mouse anti-NeuN (Abcam, Cambridge, UK), chicken anti-GFAP (Abcam, UK), anti-mouse Alexa488 (Invitrogen, Carlsbad, CA, USA), and anti-chicken Alexa647 (Invitrogen, USA). Following three additional washes with wash buffer, the cultures were stained with DAPI (diluted 1:1000, 10 min) and mounted using Fluoromount-G (Invitrogen, USA).

Confocal images were obtained using a Zeiss LSM 800 confocal laser scanning microscope (Carl Zeiss, Oberkochen, Germany).

### 4.9. RT-PCR

The total RNA from the primary cell cultures of the hippocampus was isolated on DIV21 using RNA-Extran kit (Syntol, Moscow, Russia). Transcription was performed using a commercial RT-M-MuLV-RH kit (Biolabmix, Novosibirsk, Russia) and an expression analysis was executed on a QuantStudio 5 Real-Time PCR System for Human Identification (Thermo Fisher Scientific, Waltham, USA). The following primer pairs were used for detection:

OAZ_fw: TGAGGGCAGTAAGGACAGTTT

OAZ_rev: TTCGGAGTAGGGCGGCTCT

IL1a_fw CAACCCAGATCAGCACCTTACACCTA

IL1a_rev: TCATGACAAACTTCTGCCTGACGAG

IL6_fw: AGTCCGGAGAGGAGACTTCACAGAG

IL6_rev: CACGATTTCCCAGAGAACATGTGTAA

GFAP_fw: GTTAAGCTAGCCCTGGACAT

GFAP_rev: ACAGGAATGGTGATGCGGTT

The calculation of relative mRNA levels was performed using the ∆∆Ct method.

### 4.10. Statistical Analysis

The Kruskal–Wallis non-parametric test for multiple comparisons was used to assess the statistical significance of differences between groups. Differences were considered significant at *p* ≤ 0.05.

## 5. Conclusions

In conclusion, our findings demonstrate the critical role of astrocytes in maintaining neural network function in models of amyloidosis, a key component of AD pathology. Chronic optogenetic stimulation over seven days preserved both neural network activity and its functional architecture. These results suggest that selective optogenetic activation of astrocytes may represent a promising novel therapeutic strategy for combating AD.

## Data Availability

The data used to support the findings of this study are available from the corresponding author upon request.

## Supplementary Materials

The supporting information can be downloaded at: https://www.mdpi.com/article/10.3390/ijms252212237/s1.
