# Supplementary material for: The Effect of the Optogenetic Stimulation of Astrocytes on Neural Network Activity in an In Vitro Model of Alzheimer’s Disease
Source: Int J Mol Sci. 2024 Nov 14;25(22):12237. doi: 10.3390/ijms252212237 (PMC11594756; doi:10.3390/ijms252212237)

Intact DIV14 before stimulation

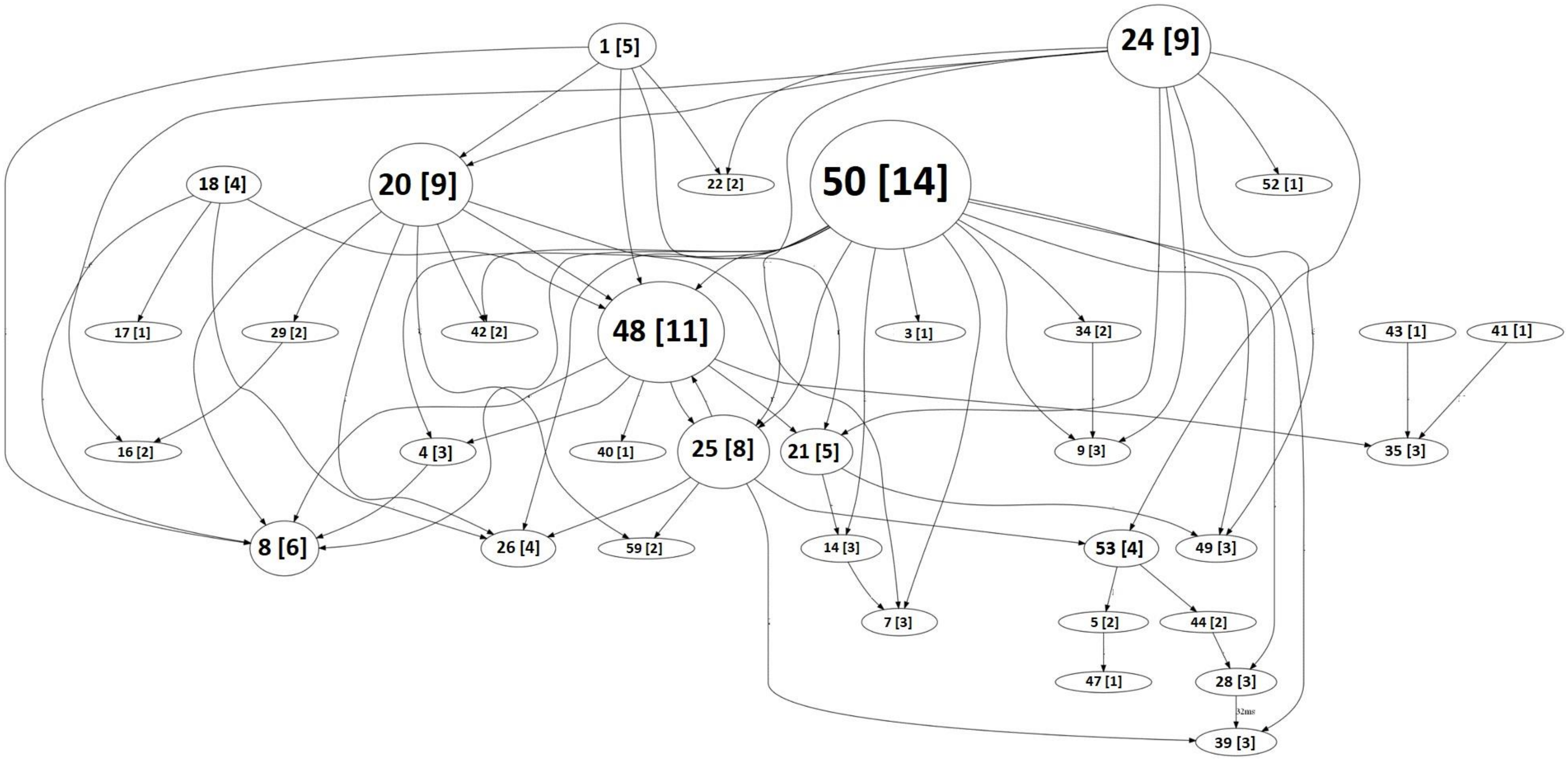

Intact DIV14 stimulation

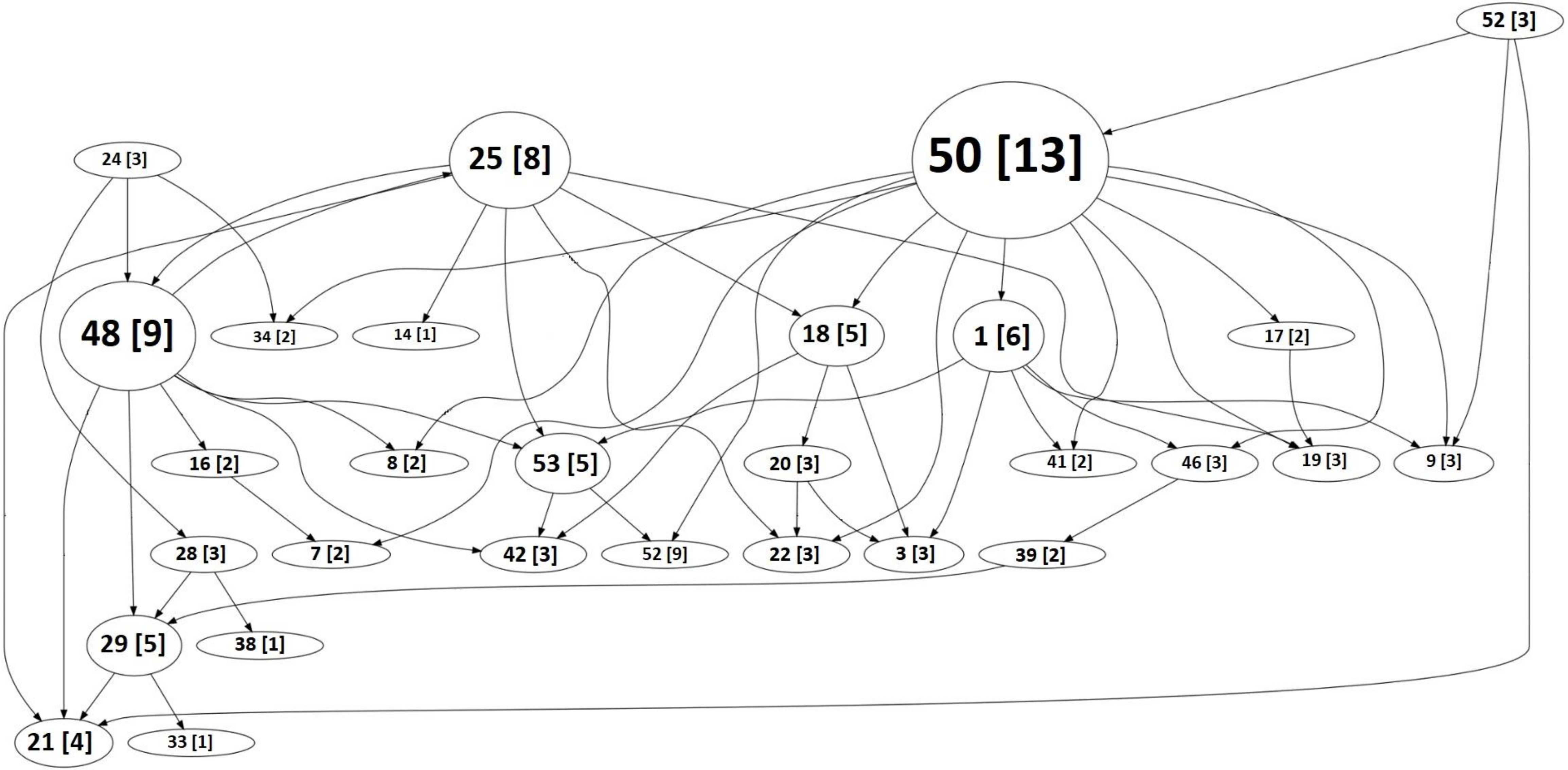

Intact DIV14 after stimulation

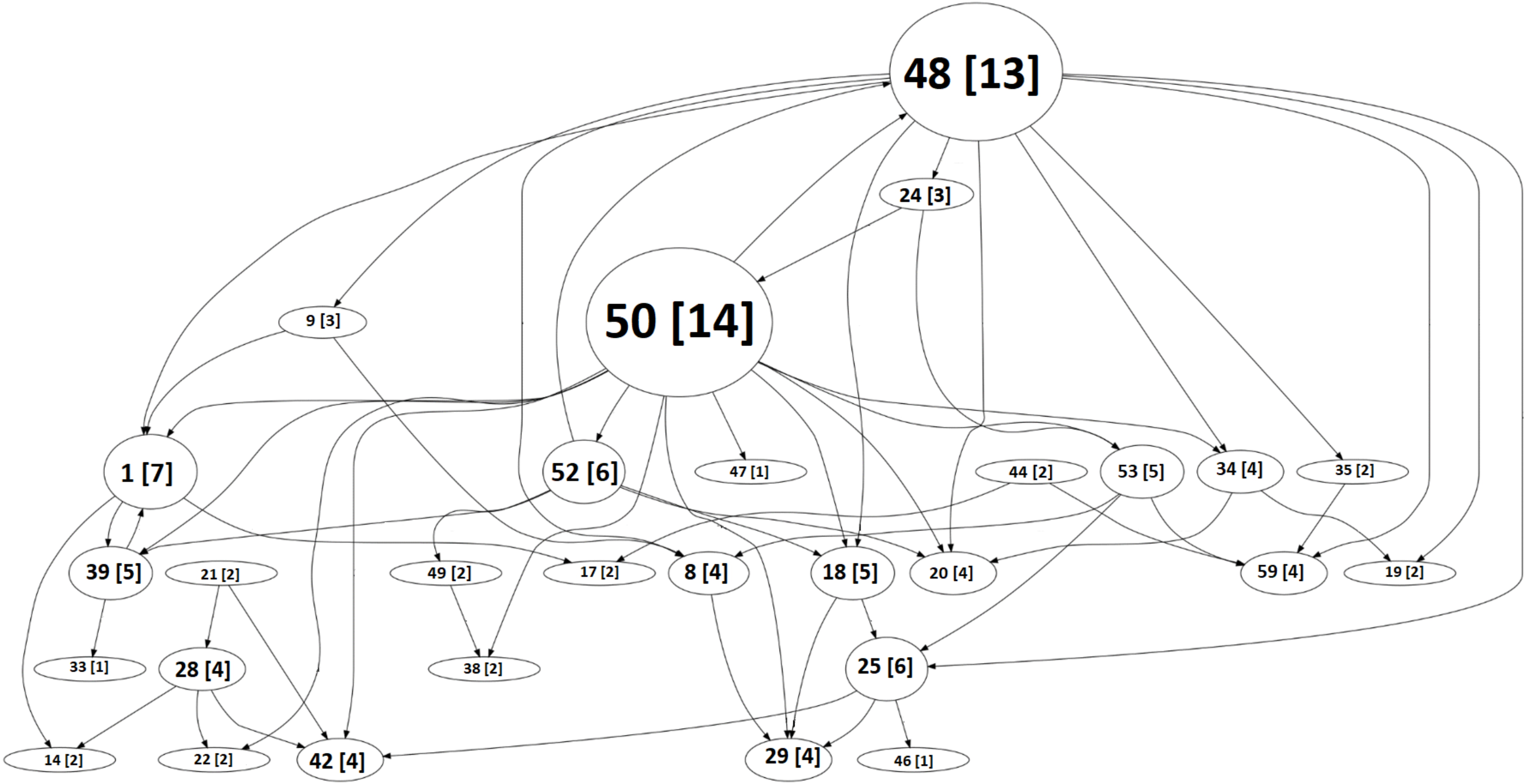

Intact DIV19

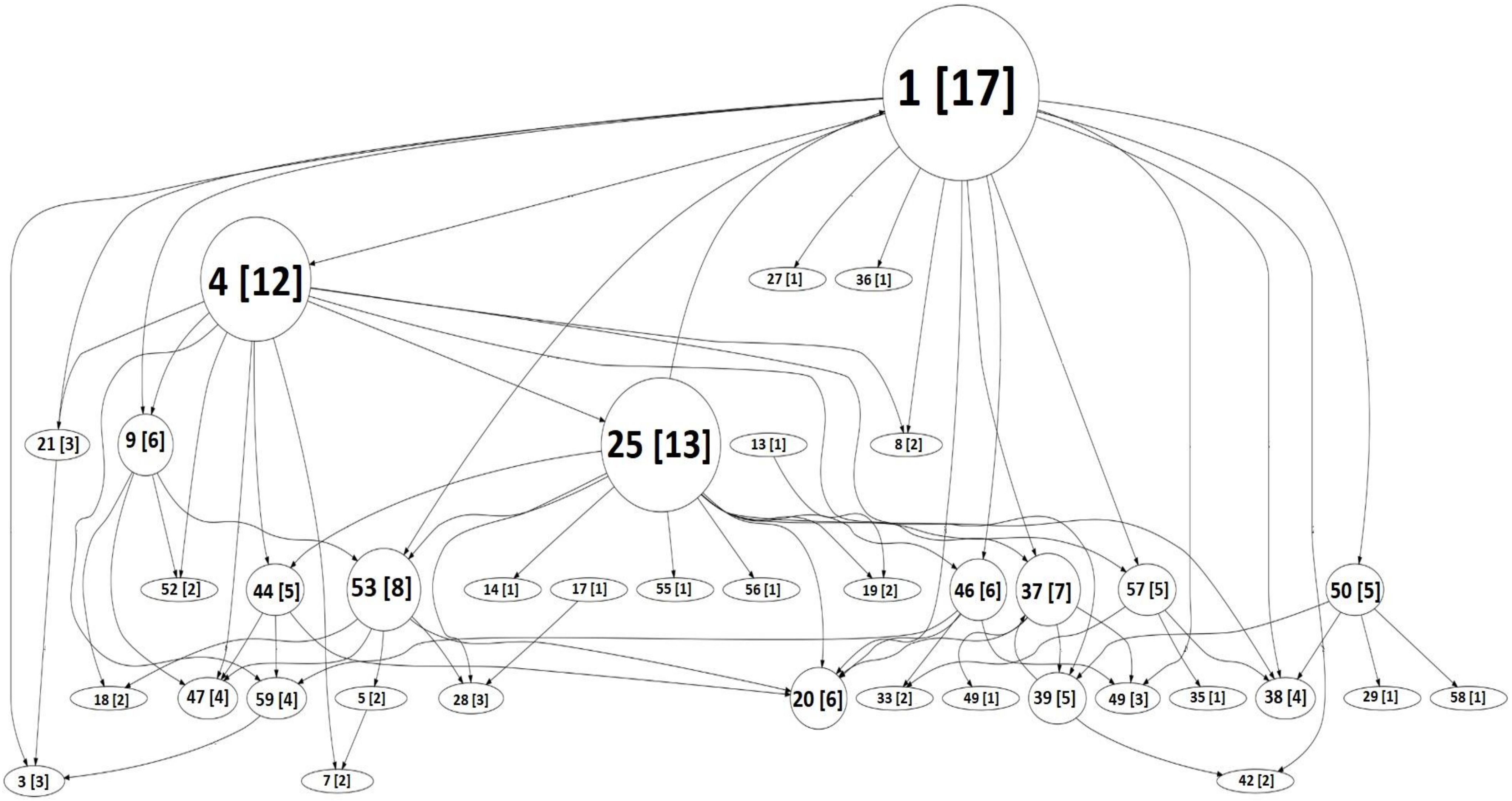

ChR2 DIV14 before stimulation

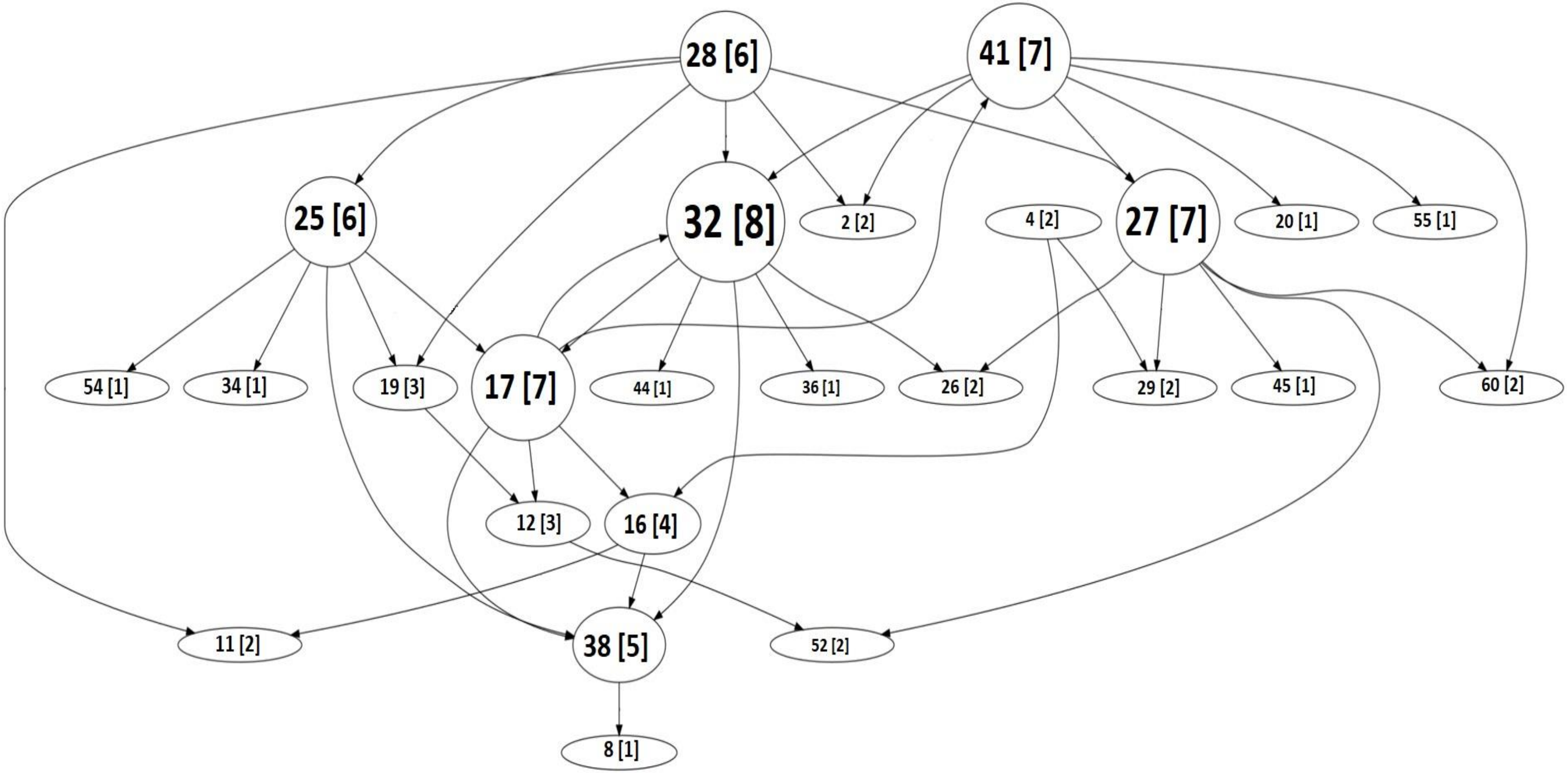

ChR2 DIV14 stimulation

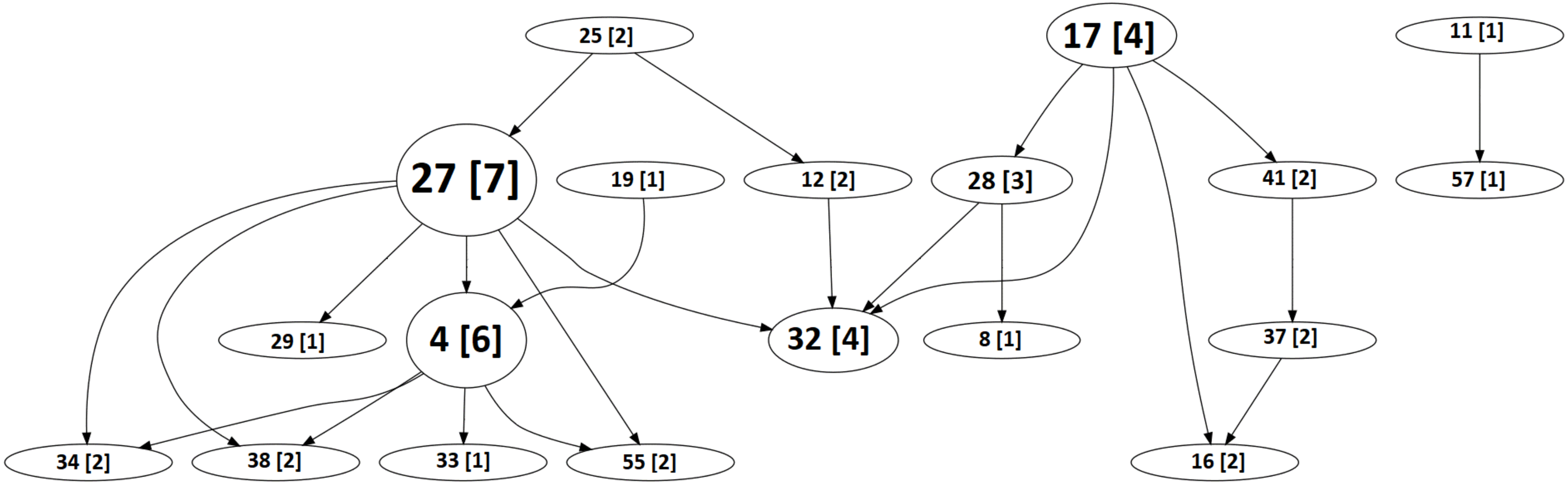

ChR2 DIV14 after stimulation

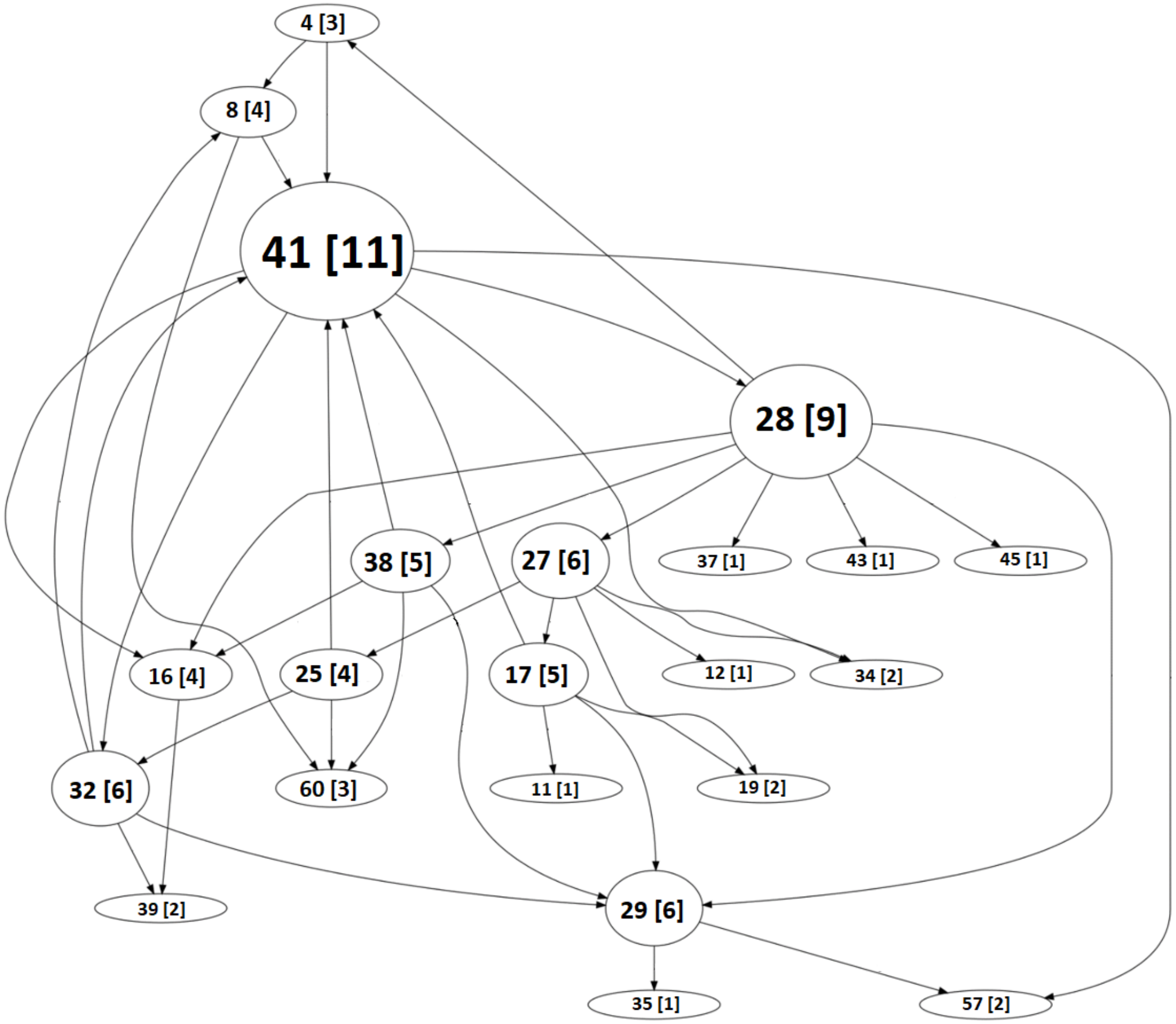

ChR2 DIV19

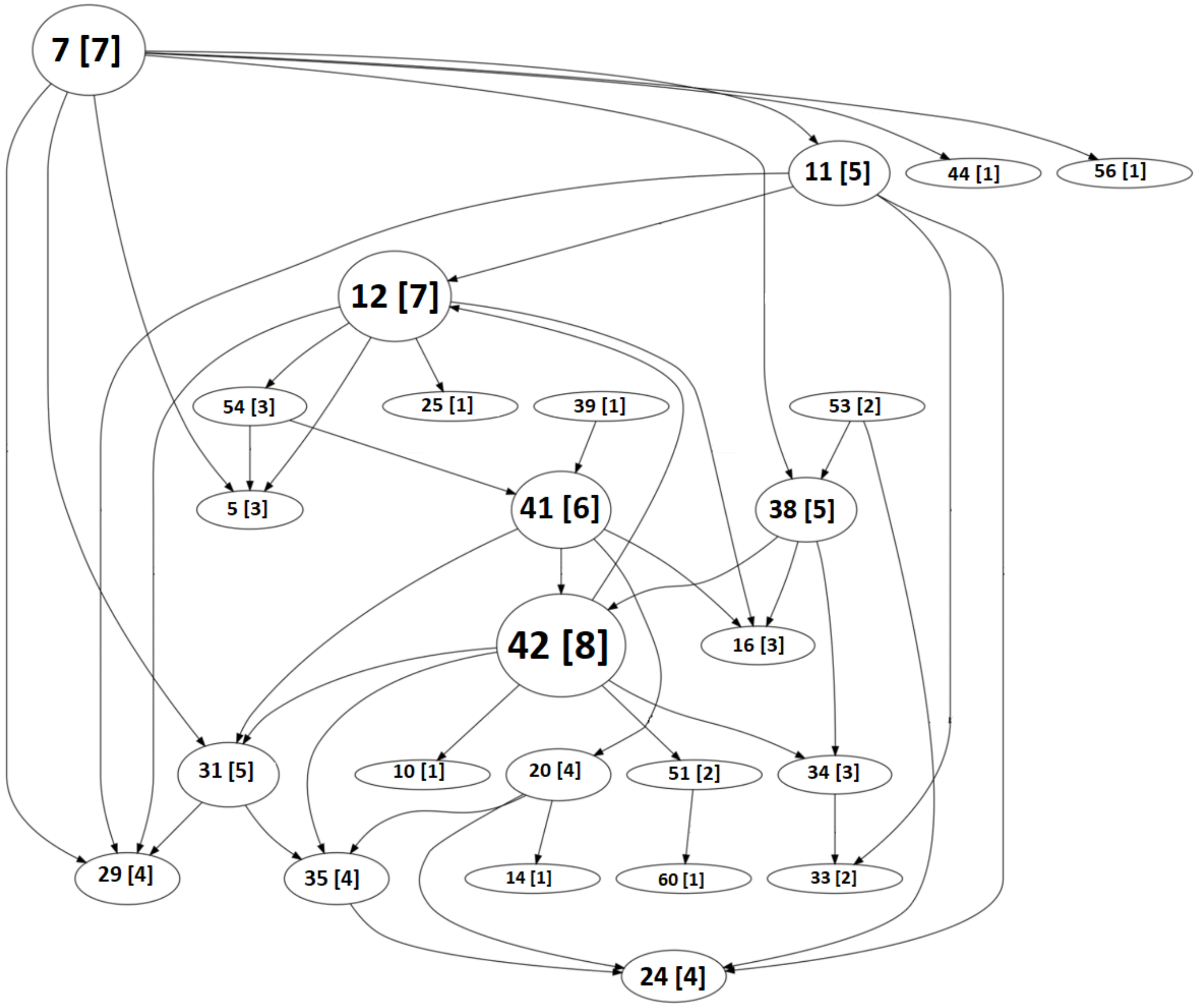

7 [7]
11 [5]
44 [1]
56 [1]
12 [7]
54 [3]
25 [1]
39 [1]
53 [2]
5 [3]
41 [6]
38 [5]
42 [8]
16 [3]
31 [5]
10 [1]
20 [4]
51 [2]
34 [3]
29 [4]
35 [4]
14 [1]
60 [1]
33 [2]
24 [4]

ChR2 chronic stimulation DIV14 before stimulation

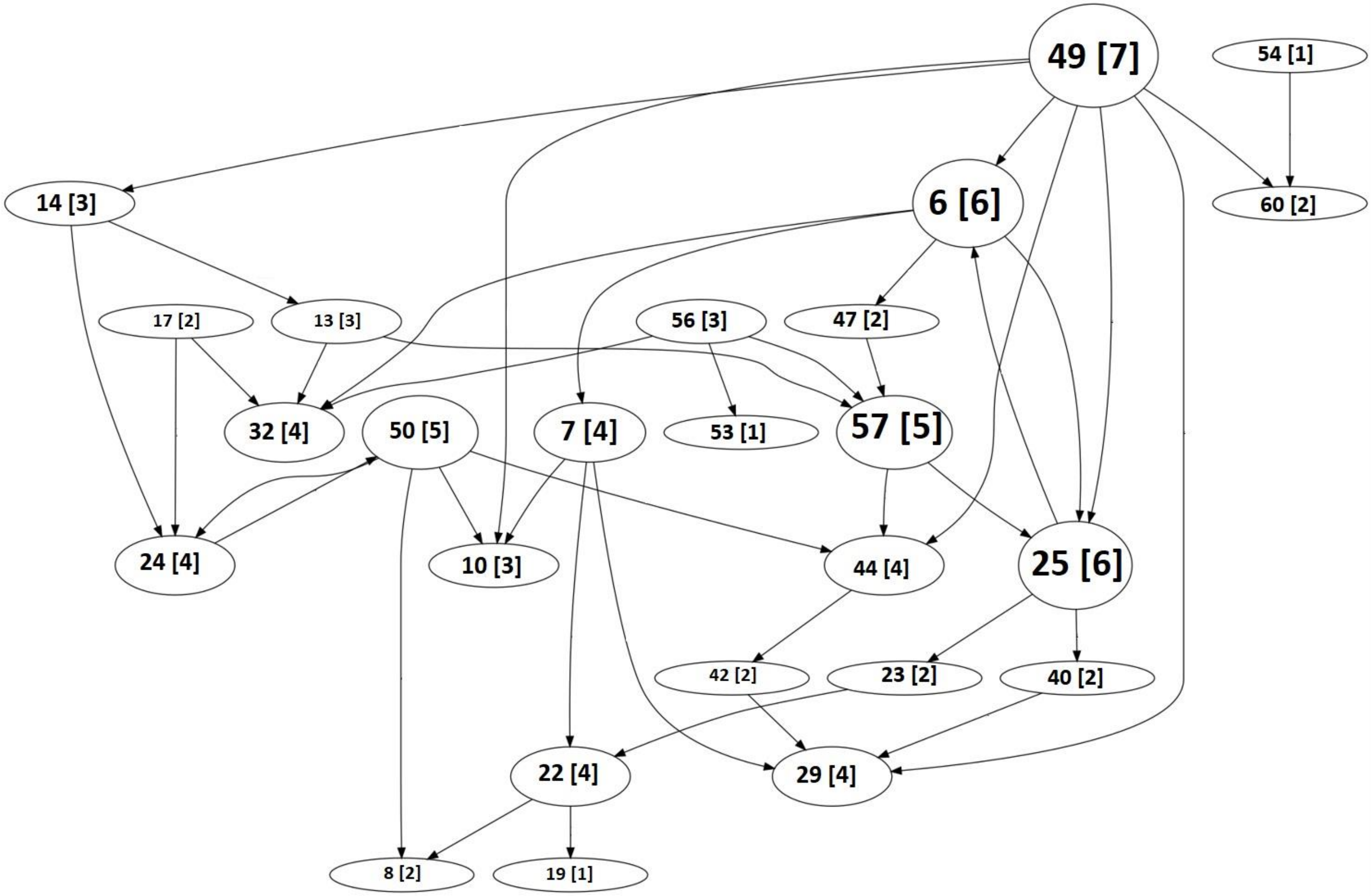

ChR2 chronic stimulation DIV14 stimulation

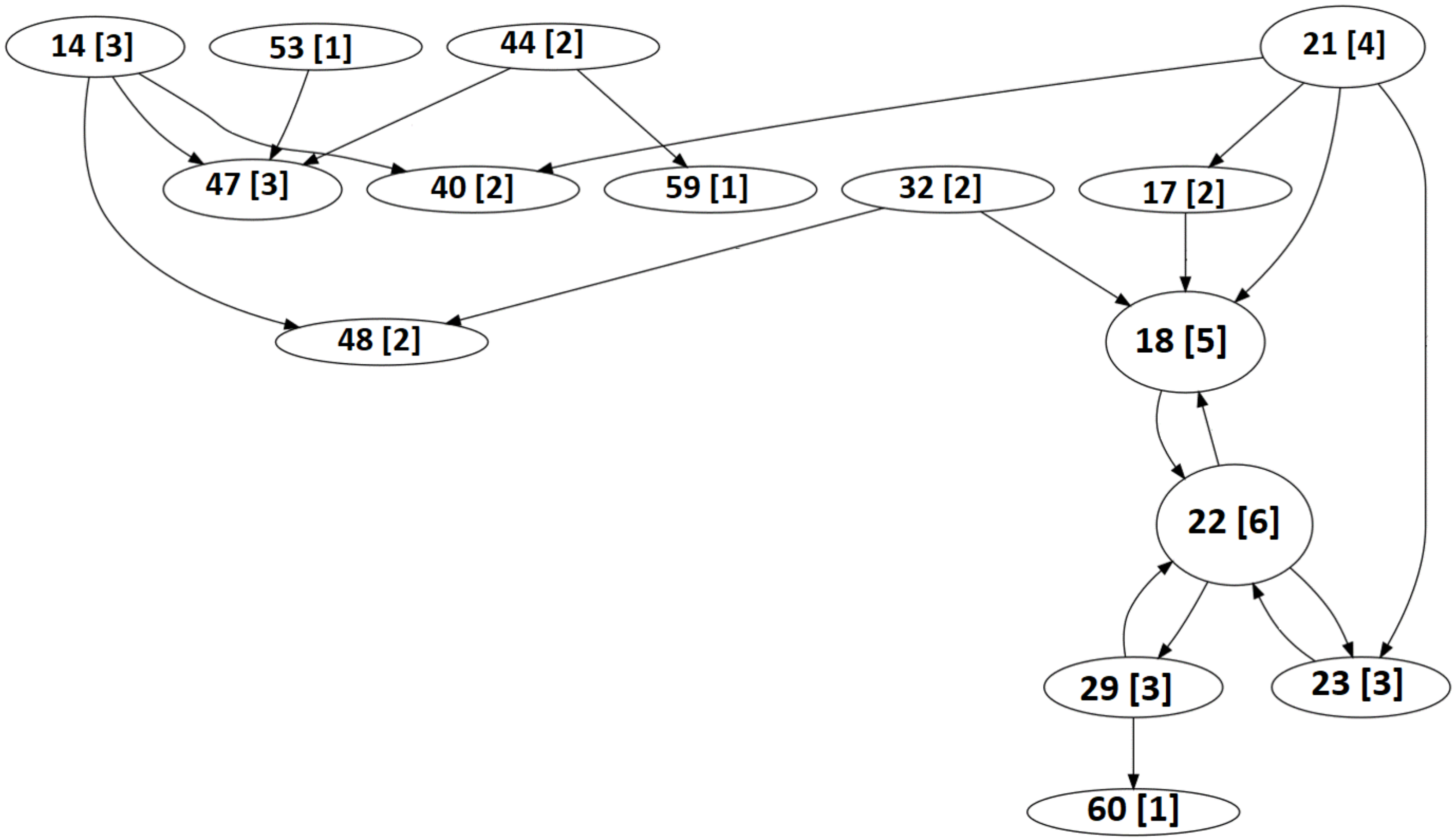

ChR2 chronic stimulation DIV14 after stimulation

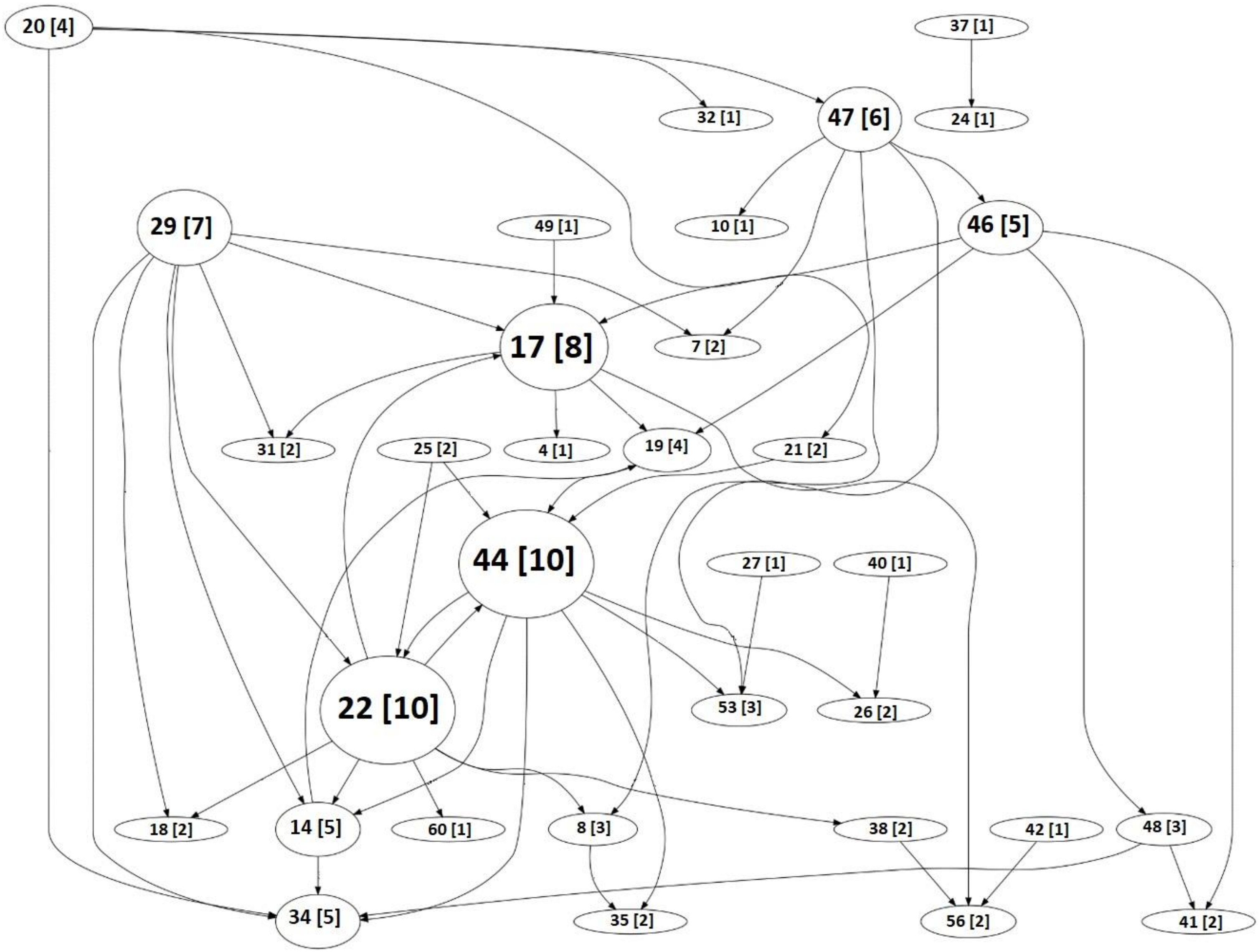

ChR2 chronic stimulation DIV19

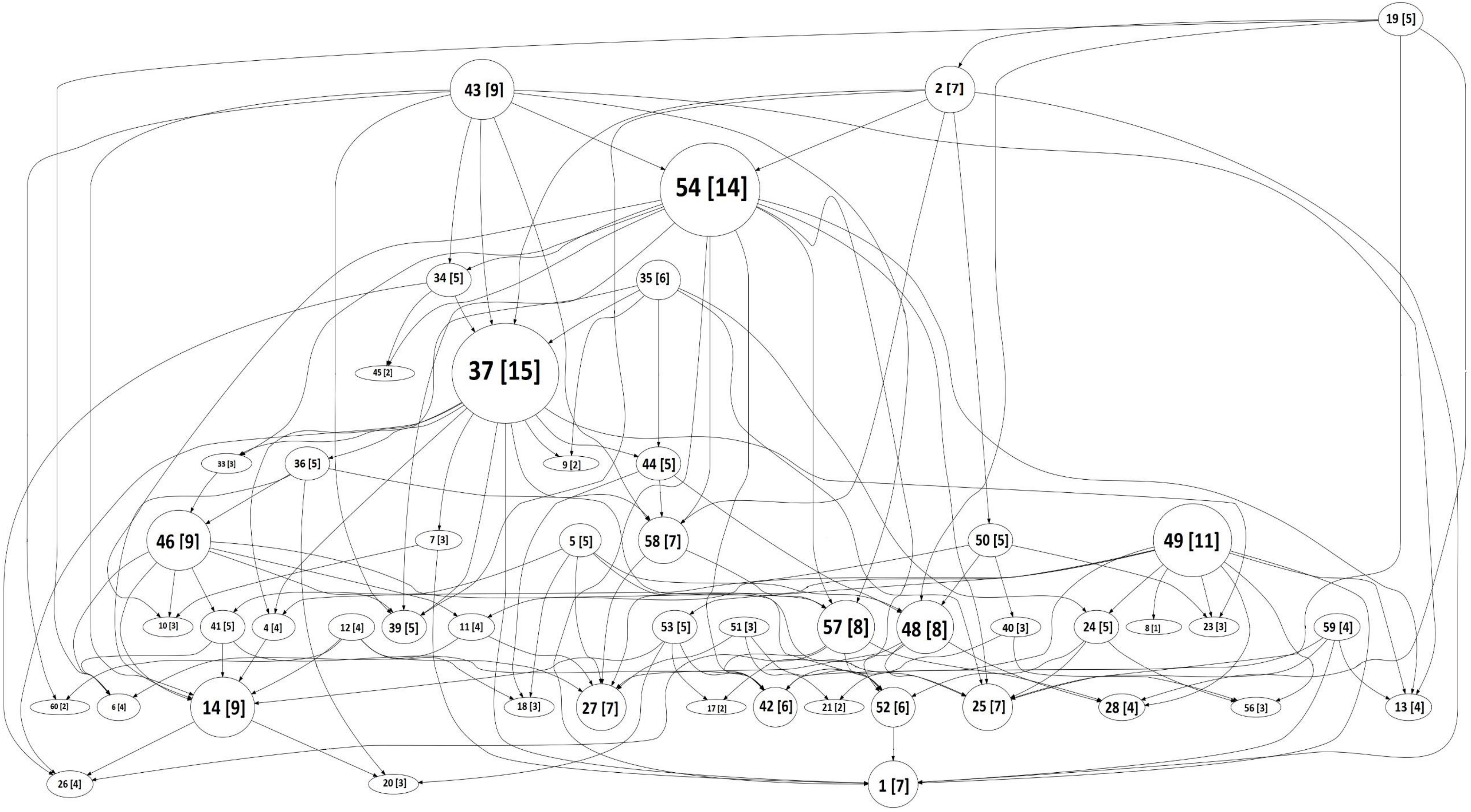

Supplement: Supplementary file 1 [file ijms-25-12237-s001.zip › graphs Figure 5.pdf]
