# Supplementary material for: The Effect of the Optogenetic Stimulation of Astrocytes on Neural Network Activity in an In Vitro Model of Alzheimer’s Disease
Source: Int J Mol Sci. 2024 Nov 14;25(22):12237. doi: 10.3390/ijms252212237 (PMC11594756; doi:10.3390/ijms252212237)
Supplement: Supplementary file 1 [file ijms-25-12237-s001.zip › Supplementary Materials_Figures_S1-S3.pdf]

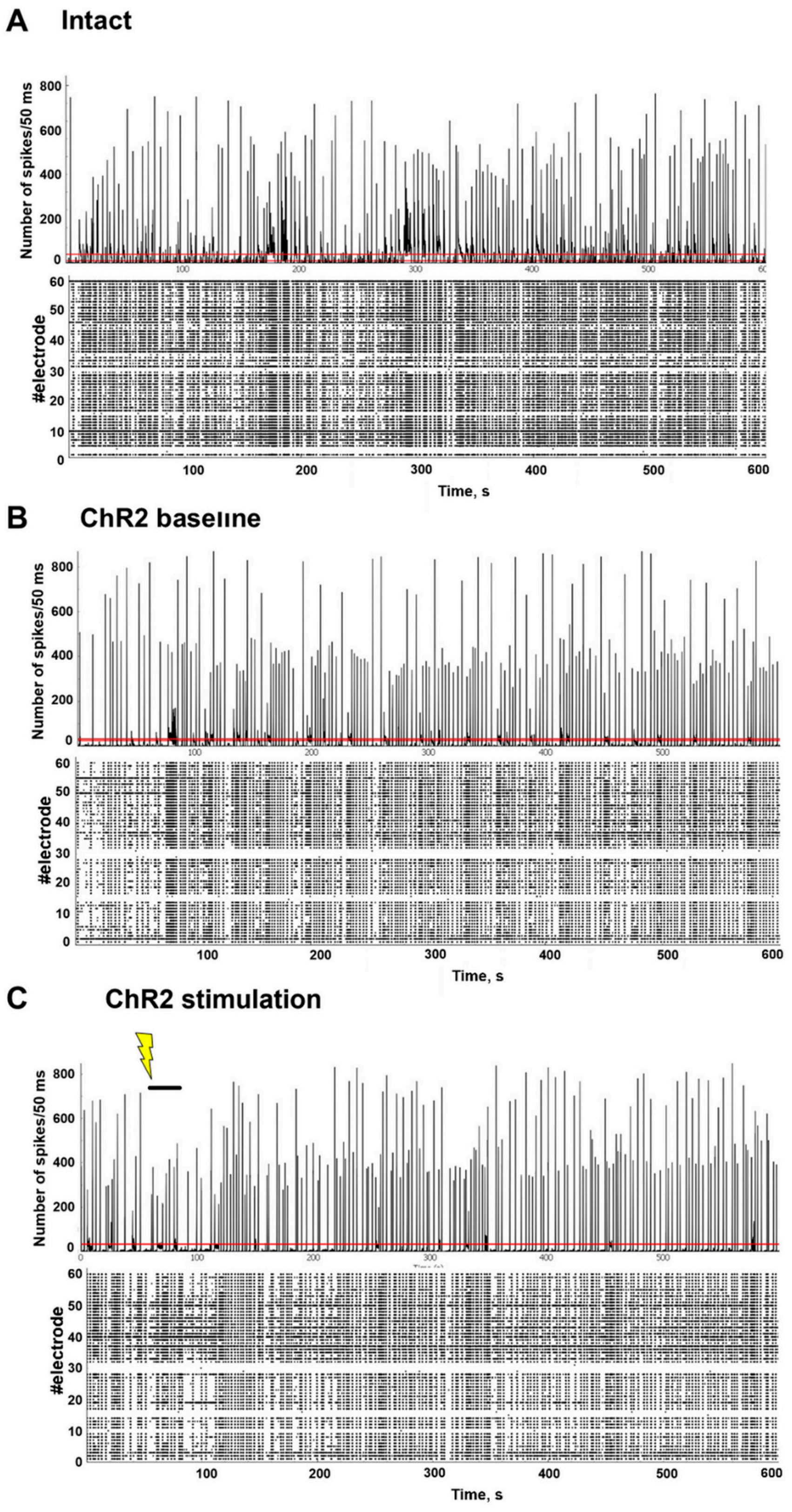

Figure S1. Number of spikes/50 ms and raster diagrams of spontaneous bioelectrical activity in primary hippocampal cultures during astrocytes optogenetic stimulation in vitro (DIV19): A – Intact culture; B - AAV-hGFAP-ChR2-EYFP-transduced culture baseline; C - AAV-hGFAP-ChR2-EYFP-transduced culture during light stimulation. Red line – threshold, yellow lightning - light stimulation 470 nm.

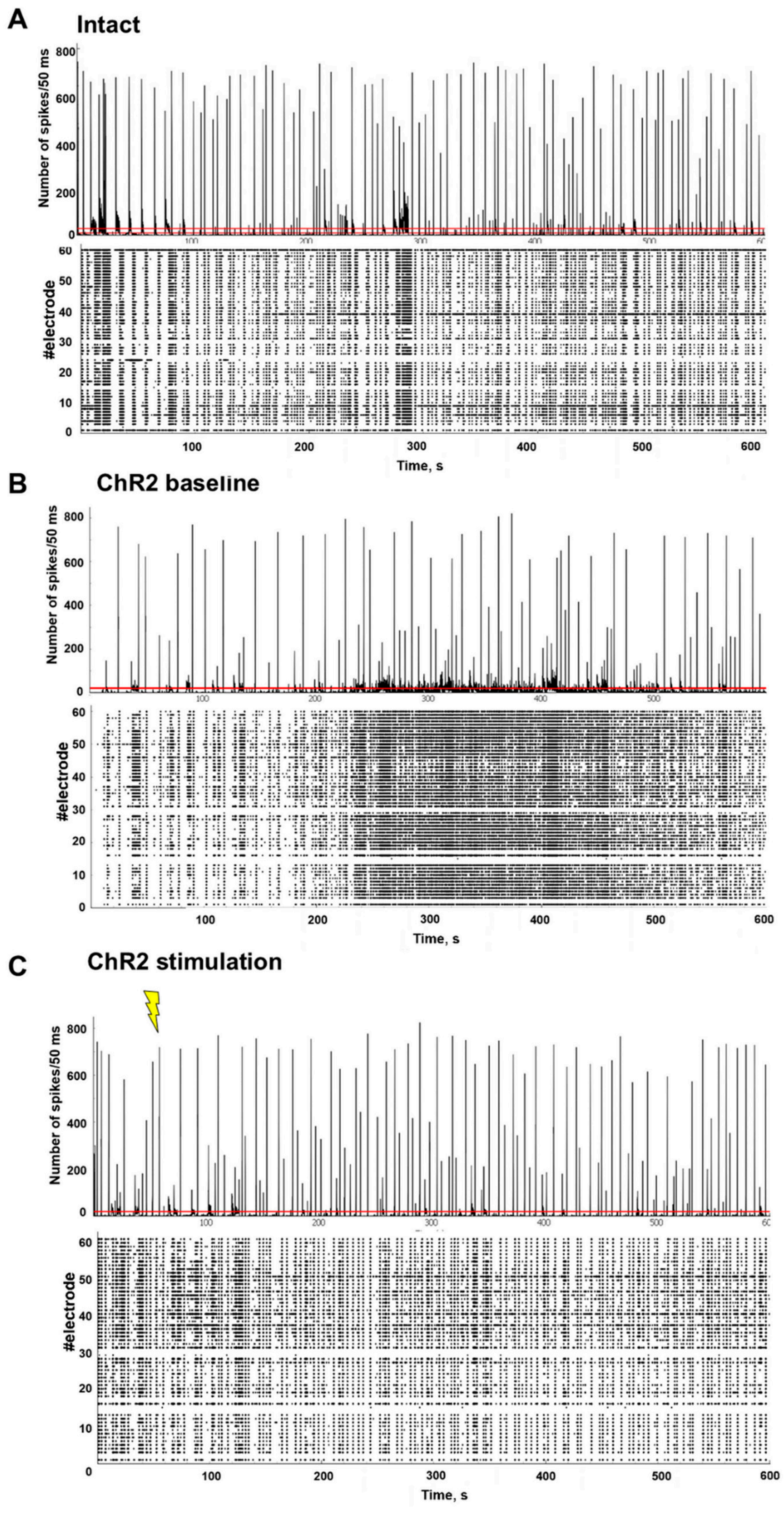

Figure S2. Number of spikes/50 ms and raster diagrams of spontaneous bioelectrical activity in primary hippocampal cultures during astrocytes optogenetic stimulation in vitro (DIV21): A – Intact culture; B - AAV-hGFAP-ChR2-EYFP-transduced culture baseline; C - AAV-hGFAP-ChR2-EYFP-transduced culture during light stimulation. Red line – threshold, yellow lightning - light stimulation 470 nm.

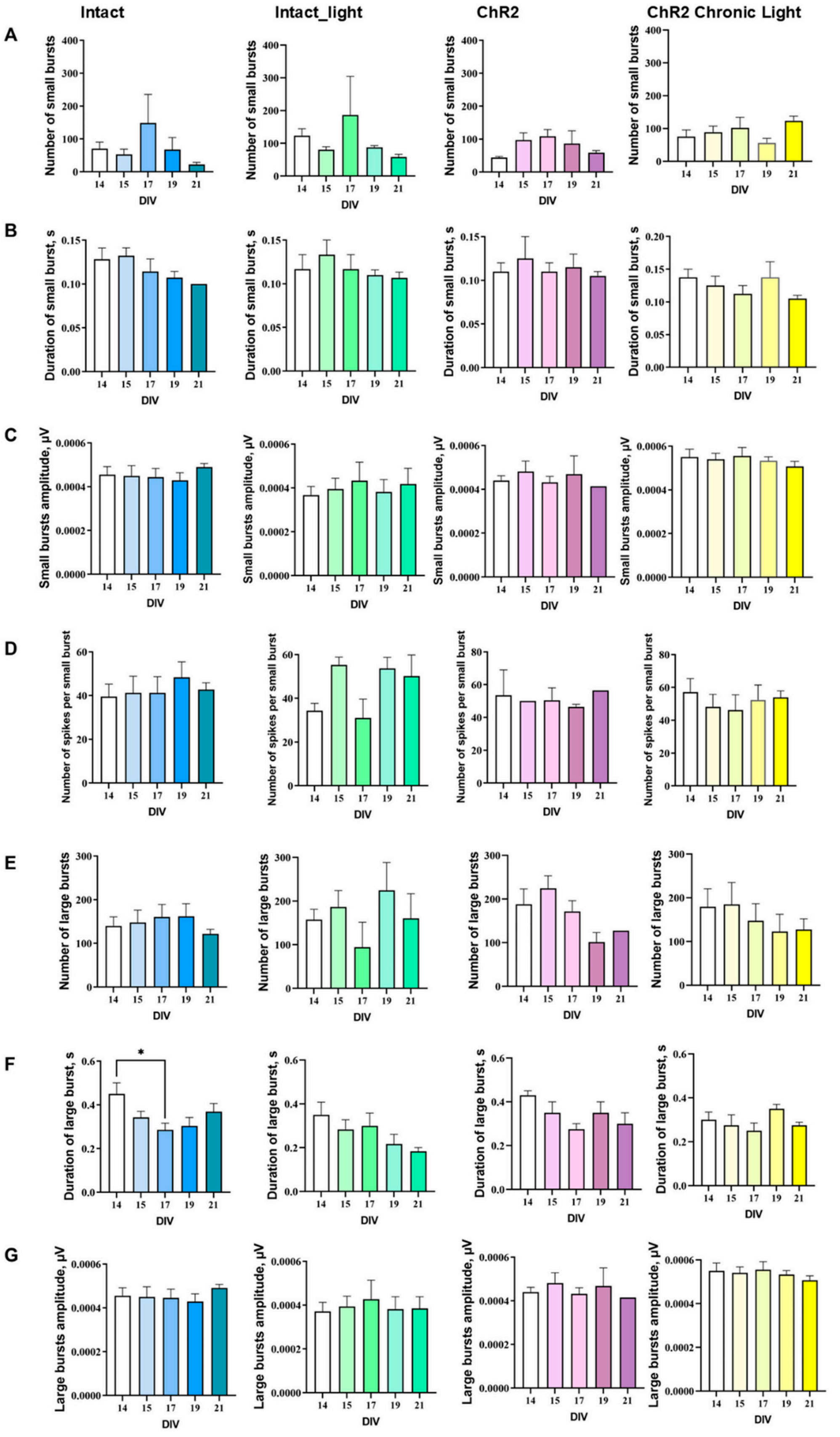

Figure S3. Parameters of spontaneous bioelectrical activity in primary hippocampal cell cultures. A - number of small bursts; B - duration of small burst, s; C - amplitude of small bursts, μV; D - Number of spikes in small burst; E - number of large bursts; F - duration of large burst, s; G - amplitude of large bursts, μV. M±SEM; ∗$p < 0.05$ - the differences are significant vs DIV14, Kruskal-Wallis test.
