# Supplementary figures and images for: The Effect of the Optogenetic Stimulation of Astrocytes on Neural Network Activity in an In Vitro Model of Alzheimer’s Disease
Source: Int J Mol Sci. 2024 Nov 14;25(22):12237. doi: 10.3390/ijms252212237 (PMC11594756; doi:10.3390/ijms252212237)

# Intact DIV14

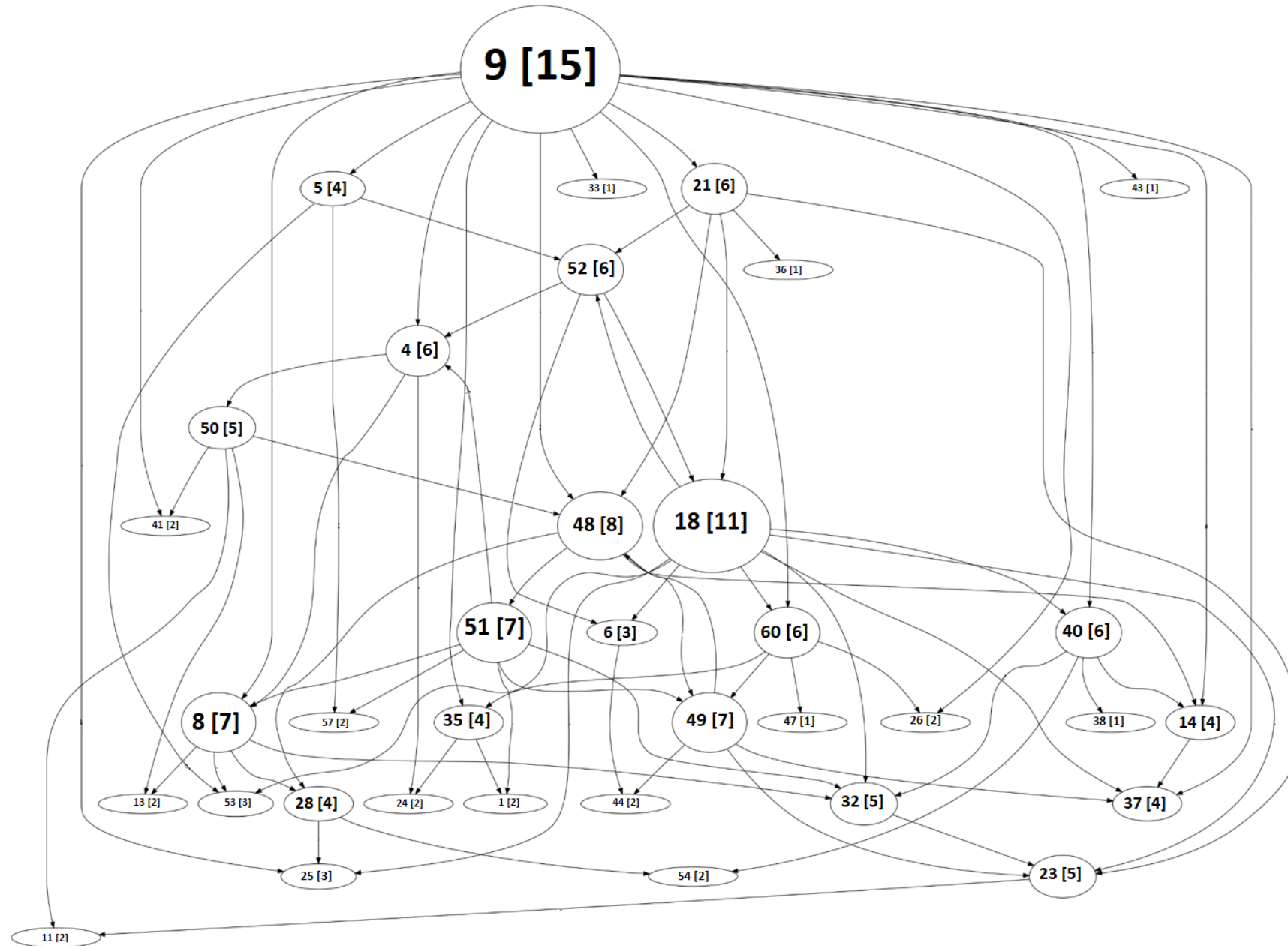

## Intact DIV19

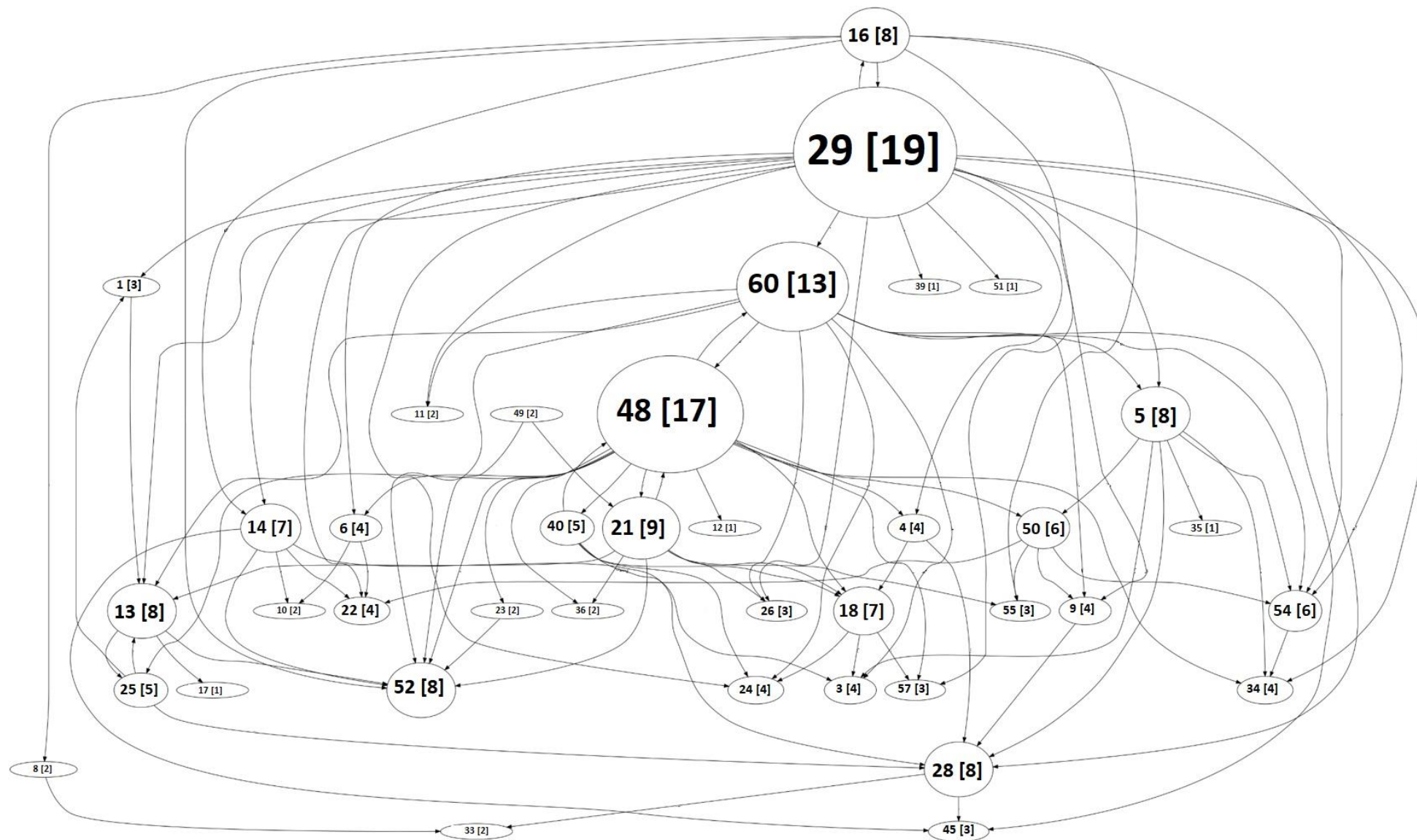

# Ab DIV14

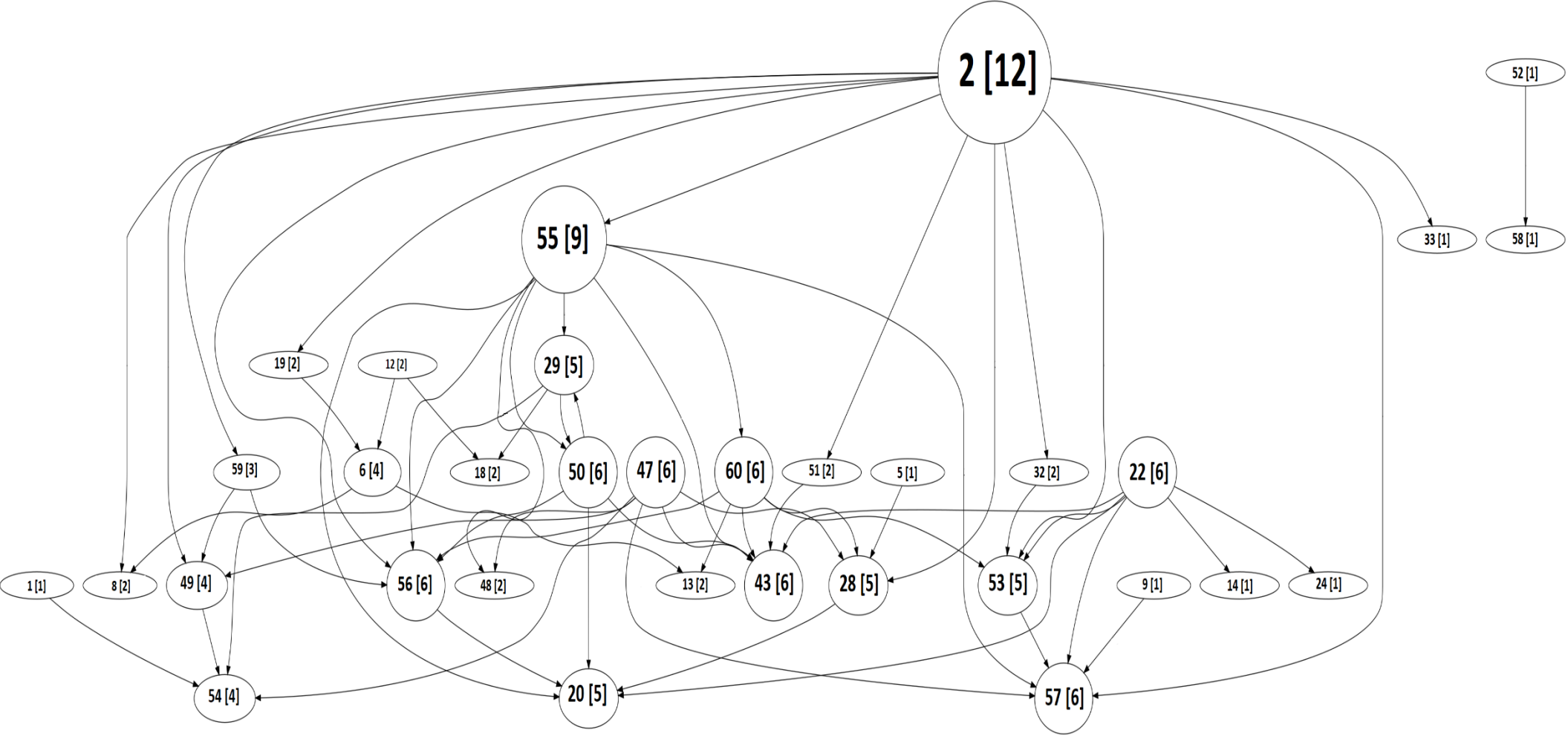

# Ab DIV19

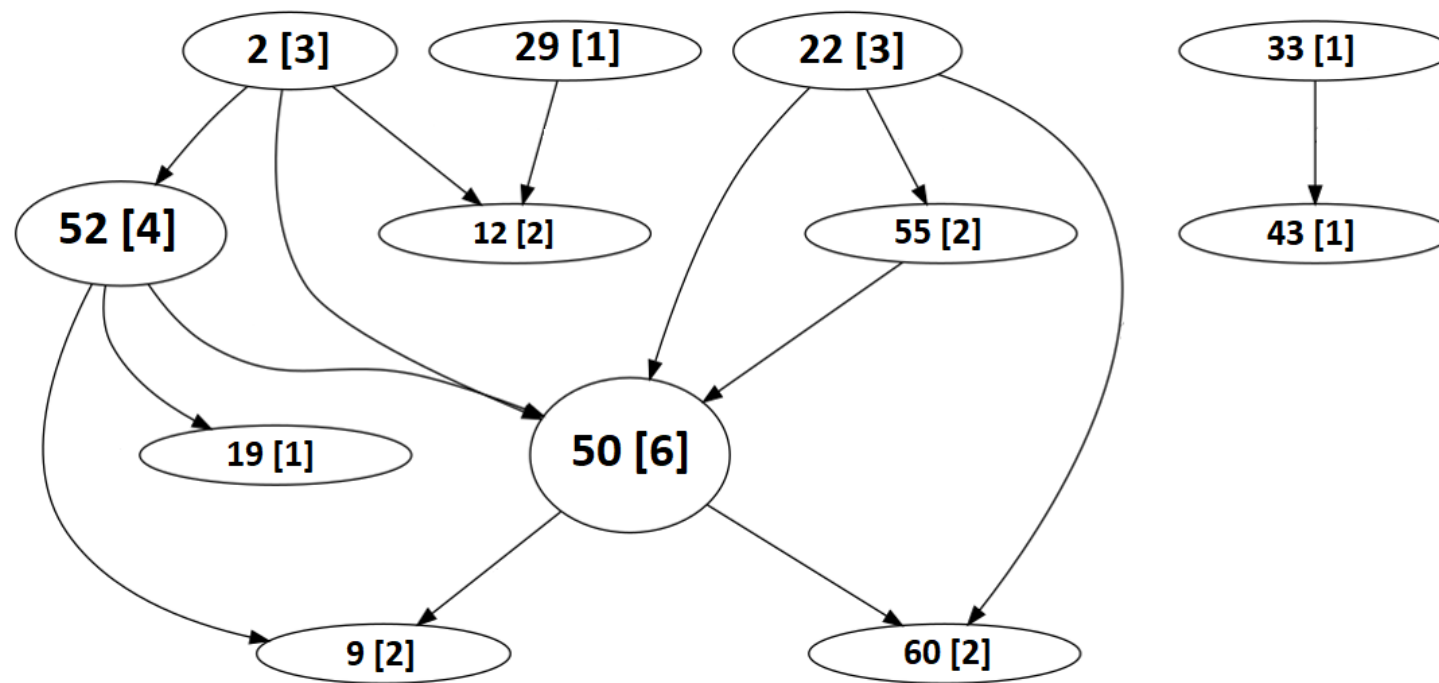



# Ab-ChR2 DIV19

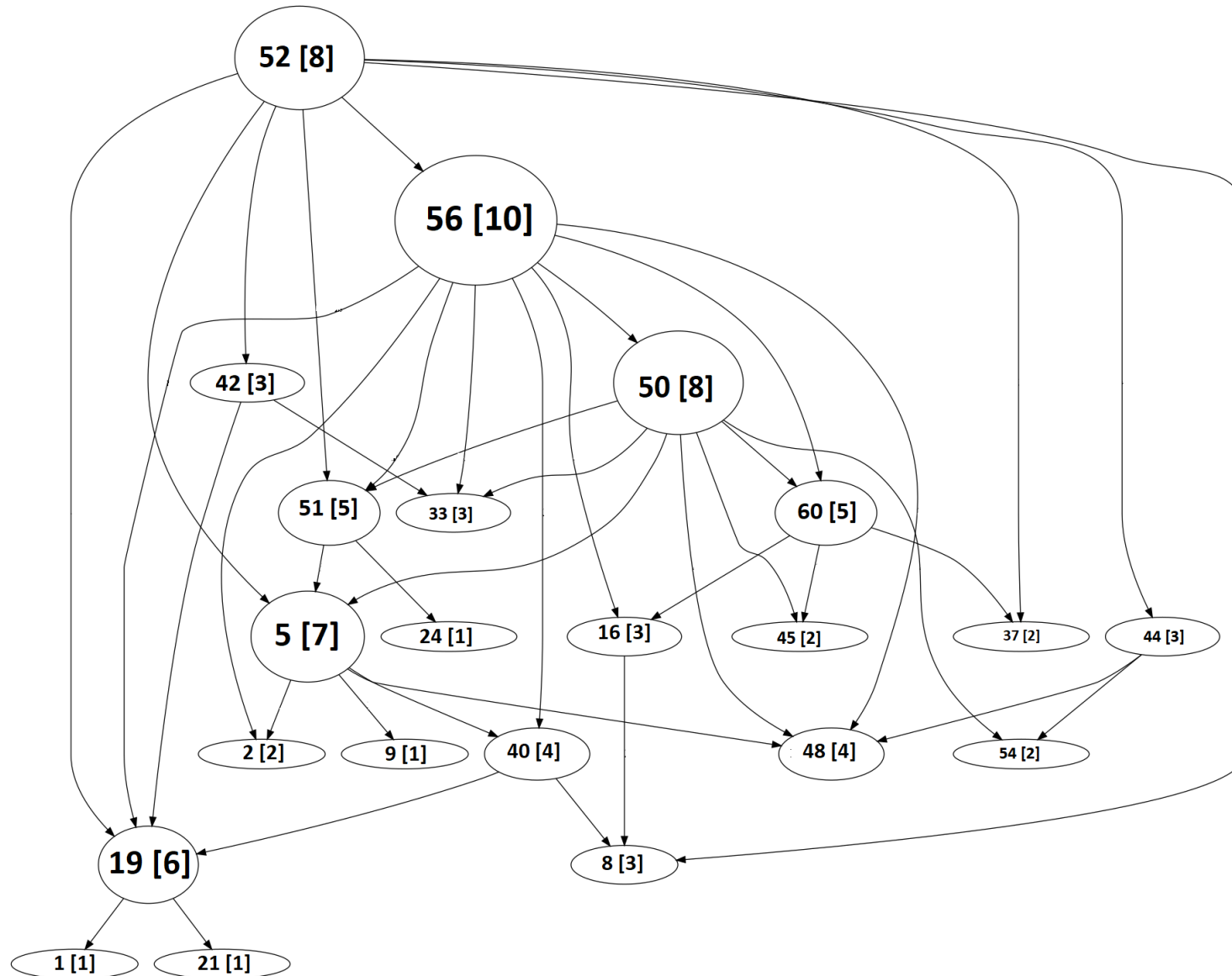

Supplement: Supplementary file 1 [file ijms-25-12237-s001.zip › graphs Figure 8.pdf]
